# Landscape of Cell States and Multicellular Ecotypes in Non-Small Cell Lung Cancer

**DOI:** 10.3390/ijms27156718

**Published:** 2026-07-28

**Authors:** Honghao Li, Yan Jiang, Zhengchun Huo, Shaokang Li, Xu Luo, Yumeng Xie, Qilemuge Xi, Shiyuan Wang, Lei Yang

**Affiliations:** 1 College of Bioinformatics Science and Technology, Harbin Medical University, Harbin 150081, China; 202501224@hrbmu.edu.cn (H.L.); jy07010701@163.com (Y.J.); h16600300977@163.com (Z.H.); 18034021182@163.com (S.L.); luoxu@hrbmu.edu.cn (X.L.); xym_2226@163.com (Y.X.); 2 The State Key Laboratory of Reproductive Regulation and Breeding of Grassland Livestock, College of Life Sciences, Inner Mongolia University, Hohhot 010070, China; qlmgxi@imu.edu.cn

**Keywords:** non-small cell lung cancer, tumor microenvironment, cell states, clinical prognosis, transcriptional regulator

## Abstract

Non-small cell lung cancer (NSCLC) is the most prevalent type of lung cancer and remains a major global health burden. Comprehensive characterization of tumor microenvironment (TME) heterogeneity is critical for understanding tumor progression and clinical outcomes in NSCLC. In this study, we identified 34 transcriptionally defined cell states and 6 multicellular ecotypes using the EcoTyper framework, thereby systematically delineating the TME landscape of NSCLC. These cell states and ecotypes were further validated across 11 independent bulk transcriptomic cohorts, one single-cell RNA sequencing cohort, and one spatial transcriptomics cohort, demonstrating strong reproducibility and robustness. Prognostic analyses revealed that several cell states and ecotypes were significantly associated with patient survival outcomes in NSCLC. Transcriptional regulatory analysis further identified *SP8*, *MAZ*, and *FOS* as potential regulators involved in shaping NSCLC ecotypes. Collectively, this study provides a comprehensive atlas of TME-associated cell states and ecotypes in NSCLC, offering new insights into tumor heterogeneity and potential opportunities for personalized prognostic assessment and therapeutic intervention.

## 1. Introduction

NSCLC is the most common type of lung cancer, accounting for approximately 80–85% of all lung cancer cases [1,2,3,4]. It is a malignant tumor originating from pulmonary epithelial cells and is characterized by substantial molecular and clinical heterogeneity [5]. NSCLC is also one of the major focuses of current precision medicine research in lung cancer. Standard treatments for patients with NSCLC include surgery, chemotherapy, radiotherapy, targeted therapy, and immunotherapy, depending on tumor stage and molecular characteristics [6,7]. Platinum-based chemotherapy drugs, such as cisplatin and carboplatin, remain commonly used for advanced NSCLC [8]. In addition, targeted therapies against *EGFR*, *ALK*, and other driver alterations, as well as immune checkpoint inhibitors targeting the PD-1/PD-L1 pathway, have significantly improved clinical outcomes and prolonged survival in selected patients [9,10]. However, owing to the pronounced clinical and molecular heterogeneity of NSCLC, patients with NSCLC often exhibit markedly diverse therapeutic responses and clinical outcomes, which substantially complicates effective disease management and personalized treatment strategies.

The TME represents a highly complex ecosystem surrounding tumor cells, comprising immune cells, stromal cells, vascular structures, and extracellular matrix components [11,12]. Accumulating evidence suggests that the TME plays a pivotal role in driving clinical heterogeneity and influencing therapeutic responses in NSCLC [13]. In recent years, the transcriptomics-based computational framework EcoTyper has emerged as a powerful tool for comprehensively dissecting the heterogeneity of the TME through the identification of distinct cell states and multicellular ecotypes [14,15,16]. By providing cell state level and ecosystem level resolution of the TME, EcoTyper facilitates a deeper understanding of tumor heterogeneity, immune regulation, disease progression, and therapeutic responses [17,18,19,20,21,22]. Increasing evidence has demonstrated that specific cell states and ecotypes are closely associated with patient prognosis, immunotherapy efficacy, and drug sensitivity, highlighting the important role of EcoTyper in precision oncology research. Therefore, it is of great importance to comprehensively characterize the TME of NSCLC at both the cell state and ecotype levels using EcoTyper framework. Such analyses may provide valuable insights into the heterogeneity and ecological organization of the TME, thereby facilitating the development of TME-targeted therapeutic strategies and advancing precision oncology research.

**Figure 1:**
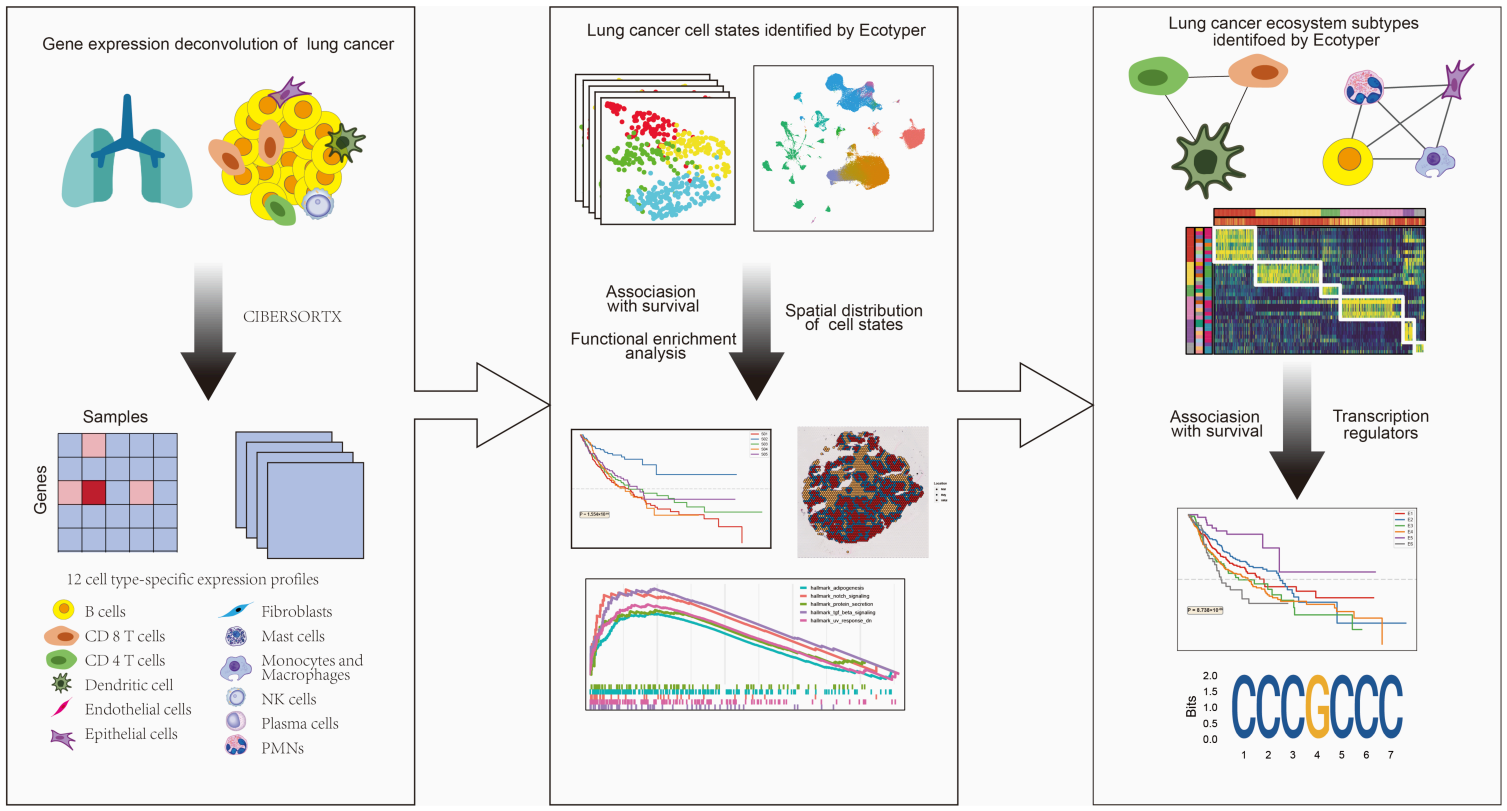
Overview of the study workflow.

In this study, we constructed an NSCLC discovery cohort comprising bulk gene expression profiles from 946 patients generated on the GPL570 platform across seven public datasets. Using EcoTyper framework, we identified 34 transcriptionally defined cell states and 6 multicellular ecotypes within the TME of NSCLC. These cell states and ecotypes defined from bulk gene expression profiles were further recovered in 11 independent bulk transcriptional validation cohorts, one scRNA-seq validation cohort, and one spatial transcriptomics validation cohort, yielding results that were consistent with those observed in the discovery cohort. Prognostic analysis revealed that most cell states and ecotypes, including epithelial cells, fibroblasts, CD4+ T cells, and endothelial cells, were significantly associated with clinical outcomes, thereby providing a prognostic atlas of cell states and ecotypes in NSCLC. In addition, several well-known transcriptional regulators, including *SP8*, *MAZ*, and *FOS*, were identified as potentially playing important roles in the ecotypes of NSCLC. Collectively, our study provides a comprehensive atlas of TME-associated cell states and ecotypes in NSCLC, offering valuable insights into the biological heterogeneity of NSCLC and potential opportunities for prognostic assessment and precision therapy. The workflow of our study was illustrated in Figure 1.

## 2. Results

### 2.1. Atlas of Transcriptionally Defined Cell States and Multicellular Ecotypes in the NSCLC Patients

**Figure 2:**
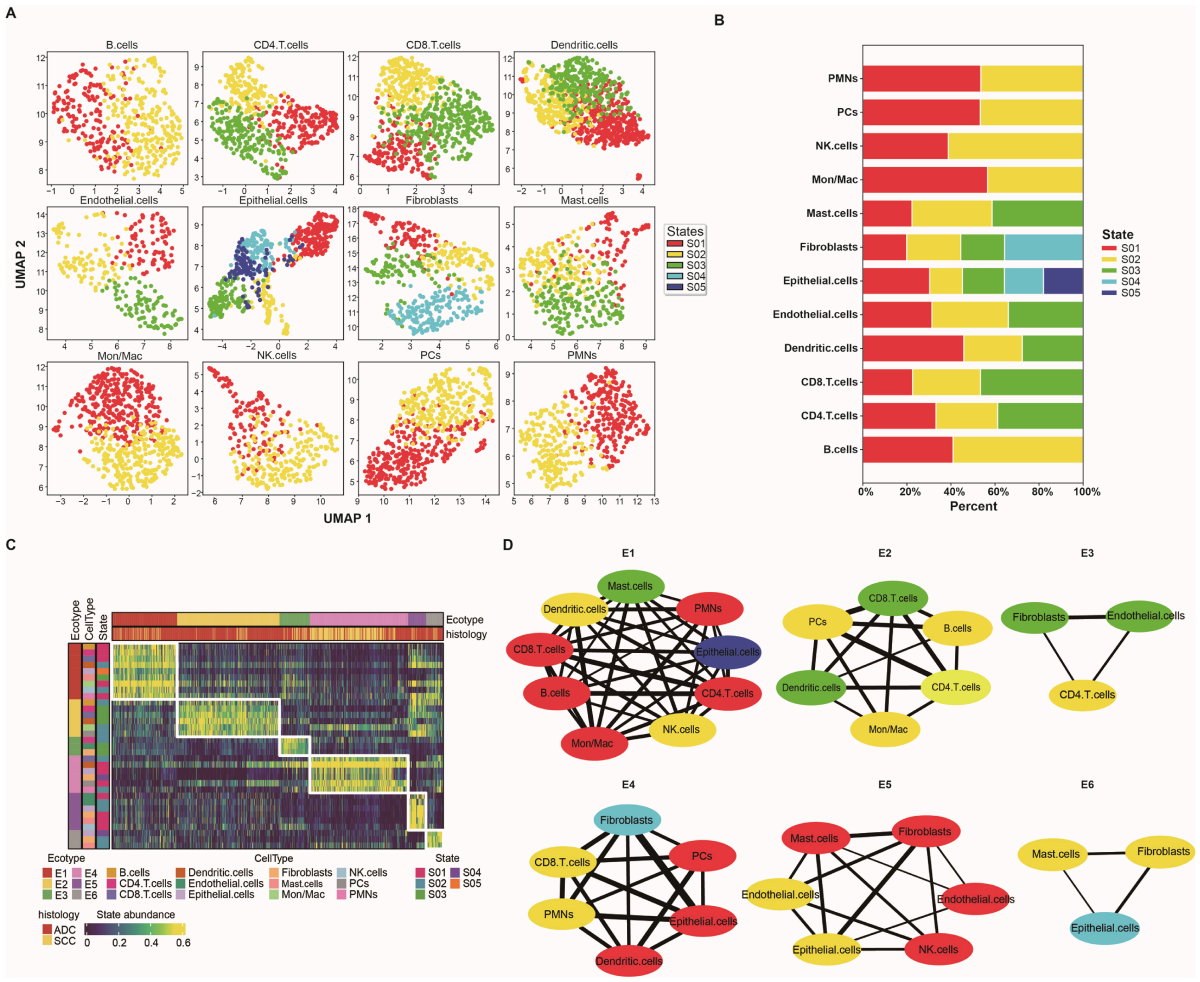
Atlas of transcriptionally defined cell states and multicellular ecotypes in NSCLC patients in the discovery cohort. (**A**) UMAP visualization of 34 cell states identified by the EcoTyper framework across 12 major cell types. Distinct cell states are indicated by different colors. (**B**) Bar plots showing the distribution of identified cell states across different cell types. Bars represent the relative proportions of each cell state within individual cell populations. (**C**) Heatmap showing the abundance patterns of cell states across six multicellular ecotypes (E1–E6) and their associations with NSCLC histological subtypes, including adenocarcinoma (ADC) and squamous cell carcinoma (SCC). (**D**) Network diagrams showing co-occurrence relationships among cell states within each ecotype. Edge thickness represents the strength of co-occurrence quantified using the Jaccard index.

To characterize transcriptionally defined cell states and multicellular ecosystems in NSCLC, EcoTyper was applied to bulk transcriptomic profiles from 946 NSCLC patients across seven GPL570 cohorts in the discovery cohort. After quality control filtering, a total of 34 distinct cell states were identified across 12 major cell types, with each cell type containing 2–5 transcriptionally distinct states (Figure 2A). Among these cellular populations, CD4+ and CD8+ T cell states were further characterized due to their established roles in antitumor immunity and clinical prognosis in NSCLC. EcoTyper identified three distinct CD4+ T cell states. State S01 exhibited high expression of several immune-associated genes, including *CD3D*, *CD2*, and *CD69*, whereas state S02 was characterized by elevated expression of genes such as *GPBP1*, *UBR1*, and *SF3B1* (Figure S1). In addition, three CD8+ T cell states were identified, with state S01 showing increased expression of cytotoxic and immune-associated genes, including *GZMB*, *GZMH*, and *CD8A* (Figure S1). Next, we investigated the distribution patterns of different cell states across NSCLC patients within each cell type, revealing substantial heterogeneity among cellular populations (Figure 2B). Most B cell and NK cell populations were assigned to state S02, whereas PMN, PC, and Mon/Mac populations displayed a more balanced distribution between states S01 and S02.

Furthermore, the distributions of cell types and cell states were compared across different histological subtypes and smoking status groups. As shown in Figure S2, significant differences were observed in three of the four distribution patterns, with the exception of cell-type distributions across smoking status groups, according to chi-square tests. These findings highlight the pronounced cellular heterogeneity among NSCLC patients. To further investigate co-occurrence relationships among distinct cell states, EcoTyper was applied to identify coherent multicellular communities, leading to the identification of six NSCLC ecotypes. Each ecotype consisted of 3–9 co-existing cell states (Figure 2C,D), reflecting structured and recurrent patterns of cellular organization within tumor samples.

Subsequently, we explored the potential biological functions of the 34 identified cell states across 12 cell types. Functional enrichment analysis based on state-specific marker genes demonstrated that these cell states were broadly associated with Hallmark and immune-related pathways (Figure S3). Across multiple cell types, significant enrichment was observed in pathways related to cytokine signaling, interferon gamma response, inflammatory response, and allograft rejection, highlighting substantial functional diversity among distinct cell populations (Figure S3A). To further illustrate state-specific functional characteristics, we focused on CD8+ T cell state S03. GO enrichment analysis demonstrated that marker genes of this state were predominantly associated with adaptive immunity and lymphocyte activation-related biological processes, including regulation of lymphocyte activation, leukocyte-mediated immunity, and adaptive immune response (Figure S3C). Consistently, pathway enrichment analysis revealed that CD8+ T cell state S03 was significantly enriched in immune-associated pathways, including interferon signaling, B-cell receptor (BCR) signaling, and T-cell receptor (TCR) signaling pathways (Figure S3D). Together, these findings suggest that this CD8+ T cell state represents an activated immune phenotype that may contribute to antitumor immune responses within the TME.

### 2.2. Recovery of Cell States and Ecotypes from External Validation Cohorts

**Figure 3:**
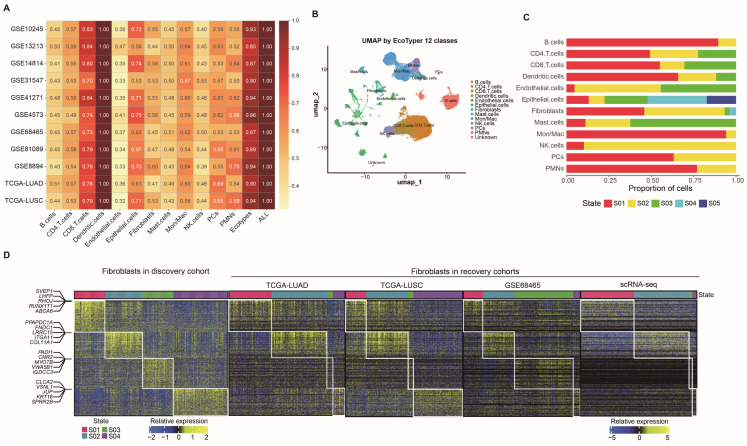
Validation of transcriptionally defined cell states and ecotypes across independent bulk transcriptomic and scRNA-seq cohorts. (**A**) Heatmap showing the proportions of patients assigned to recovered cell states and ecotypes across validation cohorts. (**B**) UMAP visualization of scRNA-seq data showing the distribution of 12 major cell types. (**C**) Bar plots showing the relative proportions of recovered cell states within each cell type in the validation scRNA-seq cohort. (**D**) Heatmap showing the four fibroblast states identified in the discovery cohort (left) and the corresponding fibroblast states recovered in validation cohorts and the scRNA-seq cohort (right).

To further validate the 34 cell states and six ecotypes identified in the discovery cohort, EcoTyper was applied to recover these cell states and ecotypes across 12 independent validation cohorts of NSCLC patients (Figure 3 and Figure S4, and Table S1). Cell states were successfully recovered in all validation cohorts, with recovery observed in 100% of patients, whereas ecotypes were recovered in 85–96% of patients across the validation cohorts (Figure 3A and Figure S4). Overall, approximately 85.03% of cell states were reproducibly and significantly identified across all validation cohorts, demonstrating the robustness and reproducibility of the cell state landscape in NSCLC (Table S1).

To further validate these findings at single-cell resolution, an independent scRNA-seq cohort of NSCLC patients was analyzed. As shown in Figure 3B, a total of 208,506 high-quality cells were successfully classified into 12 major cell types. Distinct distribution patterns of cell states were observed across these cell populations (Figure 3C). Specifically, most B cells, dendritic cells, Mon/Mac cells, PCs, and PMNs were assigned to state S01. In contrast, state S03 represented the predominant fibroblast-associated state, whereas NK cells were primarily assigned to state S02 (Figure 3C). Although some differences in cell state proportions were observed between the bulk RNA-seq discovery cohort and the scRNA-seq validation cohort, the overall distribution patterns remained largely consistent across cohorts.

To further characterize state-specific features in NSCLC, fibroblasts were selected for detailed analysis because of their critical roles in tumor initiation and progression through extracellular matrix remodeling, stromal reprogramming, and promotion of tumor growth and invasion. Four distinct fibroblast cell states were identified in the discovery cohort, and all four states were consistently recapitulated across multiple validation cohorts, including TCGA-LUAD, TCGA-LUSC, GSE68465, and the scRNA-seq cohort (Figure 3D). Moreover, highly concordant marker gene expression patterns were observed across these independent cohorts (Figure 3D). Collectively, these findings further support the robustness and reproducibility of the identified cell states across different platforms and independent NSCLC cohorts.

### 2.3. Prognostic Landscape and Therapeutic Relevance of Cell States and Ecotypes

**Figure 4:**
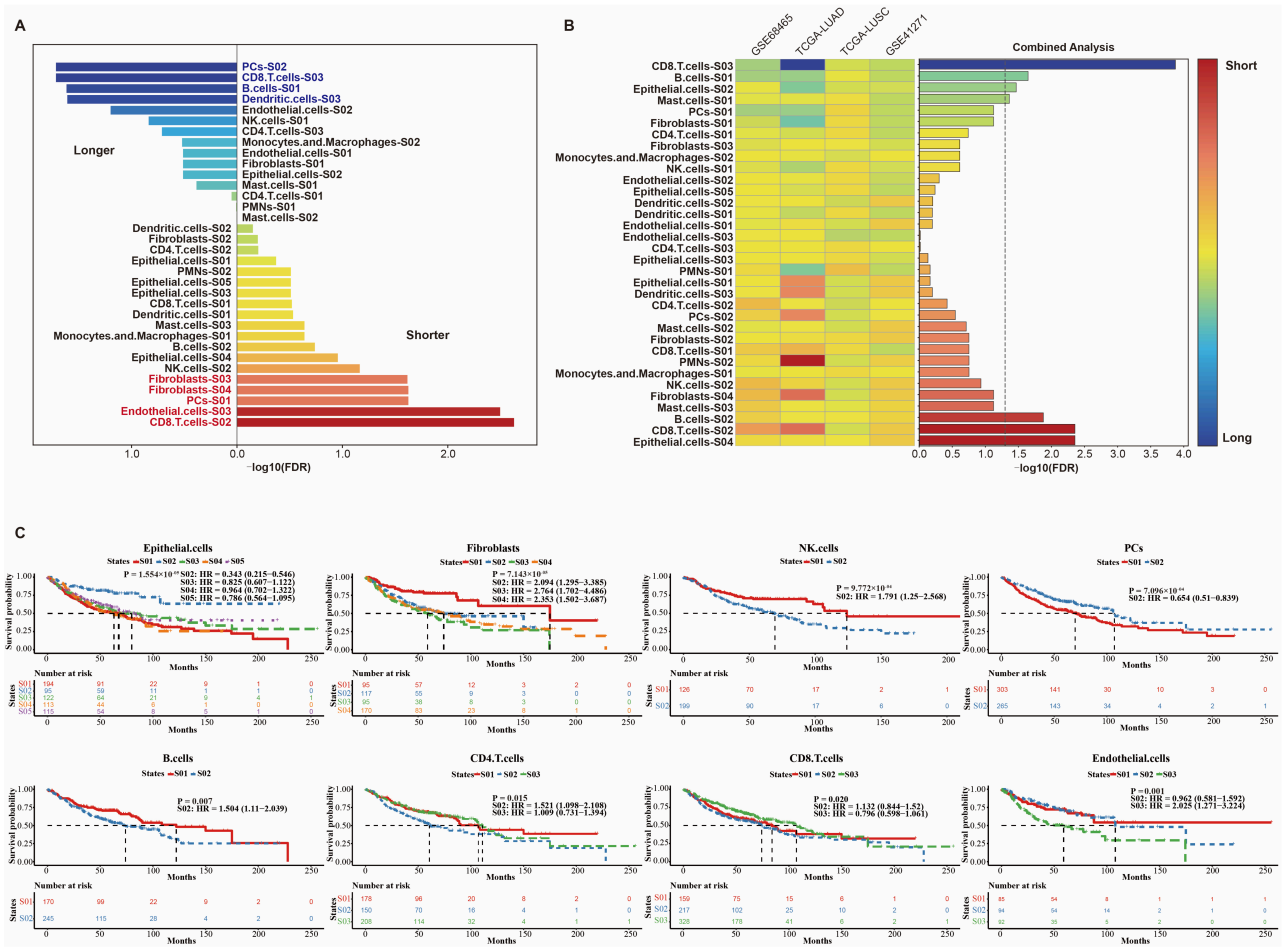
Prognostic significance of transcriptionally defined cell states in NSCLC. Associations between cell states and patient prognosis in the discovery cohort (**A**) and independent validation cohorts (**B**). Color scale represents statistical significance as −log10(FDR). (**C**) Kaplan–Meier survival analyses of representative cell states in the discovery cohort. Different colors indicate distinct cellular states, and corresponding log-rank *p*-values are shown in each panel.

Previous studies have suggested that cell states and ecotypes are closely associated with clinical outcomes across multiple cancer types. To systematically characterize their prognostic relevance in NSCLC, we comprehensively evaluated the associations between 34 cell states, six ecotypes, and patient survival outcomes. Univariable Cox regression analysis identified 9 cell states significantly associated with overall survival in the discovery cohort (Figure 4A). Among these, four cell states, including CD8+ T cell state S03, PC state S02, B cell state S01, and dendritic cell state S03, were associated with favorable prognosis. In contrast, five cell states, including CD8+ T cell state S02, endothelial cell state S03, PC state S01, fibroblast states S04 and S03, were significantly associated with poorer prognosis (Figure 4A). These prognostic associations were further validated across independent cohorts, where broadly consistent survival patterns were observed (Figure 4B). We next evaluated survival differences among patients across epithelial cell states in the discovery cohort. Significant differences in overall survival were observed among the five epithelial cell states (log-rank test, *p*-value = 1.55 × 10^−5^), with epithelial cells S02 exhibiting the most favorable prognosis (Figure 4C). Similar survival differences were also observed across fibroblasts, NK cells, PCs, B cells, CD4+ T cells, CD8+ T cells, and endothelial cells (Figure 4C).

We further investigated the prognostic significance of multicellular ecotypes in NSCLC. Significant survival differences among the six ecotypes were observed in both the discovery cohort (Figure S5A; log-rank test, *p*-value = 2.99 × 10^−5^) and the TCGA-LUAD validation cohort (Figure S5B; log-rank test, *p*-value = 3.17 × 10^−6^). Univariable Cox regression analysis identified four ecotypes significantly associated with prognosis, among which E2 and E5 were associated with favorable survival, whereas E4 and E6 were associated with poorer clinical outcomes (Figure S5C). Functional enrichment analysis of ecotype-specific upregulated genes revealed distinct biological characteristics among ecotypes. Genes upregulated in E4 were significantly enriched in pathways related to allograft rejection, inflammatory response, and epithelial–mesenchymal transition (EMT), whereas genes highly expressed in E5 were enriched in cytokine signaling and MYOGENESIS-related pathways (Figure S5C). A multivariable Cox proportional hazards model was also fitted for prognostic analysis. The global likelihood ratio test for the full model was statistically significant (*p*-value = 3.22 × 10^−3^), indicating that the model provides significant prognostic information (Figure S6).

To further investigate the prognostic significance of cell state-associated genes, we constructed a prognostic risk score model based on marker genes derived from cell states. A total of 823 marker genes were initially identified, among which 111 genes were significantly associated with overall survival according to univariable Cox regression analysis (log-rank test, *p*-value < 0.05). Subsequently, LASSO Cox regression analysis identified a 42-gene prognostic signature for model construction. The discovery cohort was used for model training, whereas the TCGA cohort served as an independent validation cohort. This design was adopted because the prognostic marker genes were initially identified from the discovery cohort, making it the appropriate dataset for model development. In contrast, the TCGA cohort was selected for validation owing to its comprehensive clinical annotations and extensive molecular characterization, which enabled a more robust evaluation of the model’s prognostic performance and generalizability. Patients were stratified into high- and low-risk groups using a predefined cutoff derived from the median risk score of the discovery cohort (0.899). This cutoff was consistently applied to both the discovery and TCGA cohorts without recalculation in the validation cohort, thereby enabling an unbiased assessment of model generalizability. As shown in Figure S7A,B, the optimal λ value was determined based on LASSO coefficient profiles and cross-validation analysis. Kaplan–Meier survival analysis demonstrated significantly poorer overall survival among high-risk patients compared with low-risk patients in both the discovery cohort (Figure S7C; log-rank test, *p*-value = 4.83 × 10^−20^) and the TCGA validation cohort (Figure S7D; log-rank test, *p*-value = 3.74 × 10^−6^). Time-dependent ROC analysis showed AUC values of 0.672, 0.715, and 0.712 for 1-, 3-, and 5-year survival prediction in the discovery cohort (Figure S7E), and 0.658, 0.628, and 0.617 in the TCGA cohort (Figure S7F), respectively. The C-index was 0.666 (95% CI, 0.640–0.692) in the discovery cohort and 0.624 (95% CI, 0.576–0.671) in the TCGA cohort, indicating consistent prognostic discrimination of the risk score model across independent cohort.

Finally, associations between cell states and therapeutic drug sensitivity were evaluated using the oncoPredict framework in the TCGA cohort. Significant associations between cell states and predicted sensitivity to multiple chemotherapeutic and targeted agents were observed, suggesting potential clinical relevance of cell states in guiding therapeutic strategies for NSCLC patients (Figure S8).

### 2.4. Transcriptional Regulatory Landscape Across Distinct Ecotypes

To further investigate the molecular mechanisms underlying distinct ecotypes in NSCLC, we analyzed transcriptional regulatory programs associated with ecotype-specific upregulated genes. By integrating transcription factor (TF) binding motif enrichment analysis with ecotype-associated gene signatures, distinct regulatory patterns were identified across ecotypes, suggesting diverse molecular drivers underlying these multicellular ecosystems.

**Figure 5:**
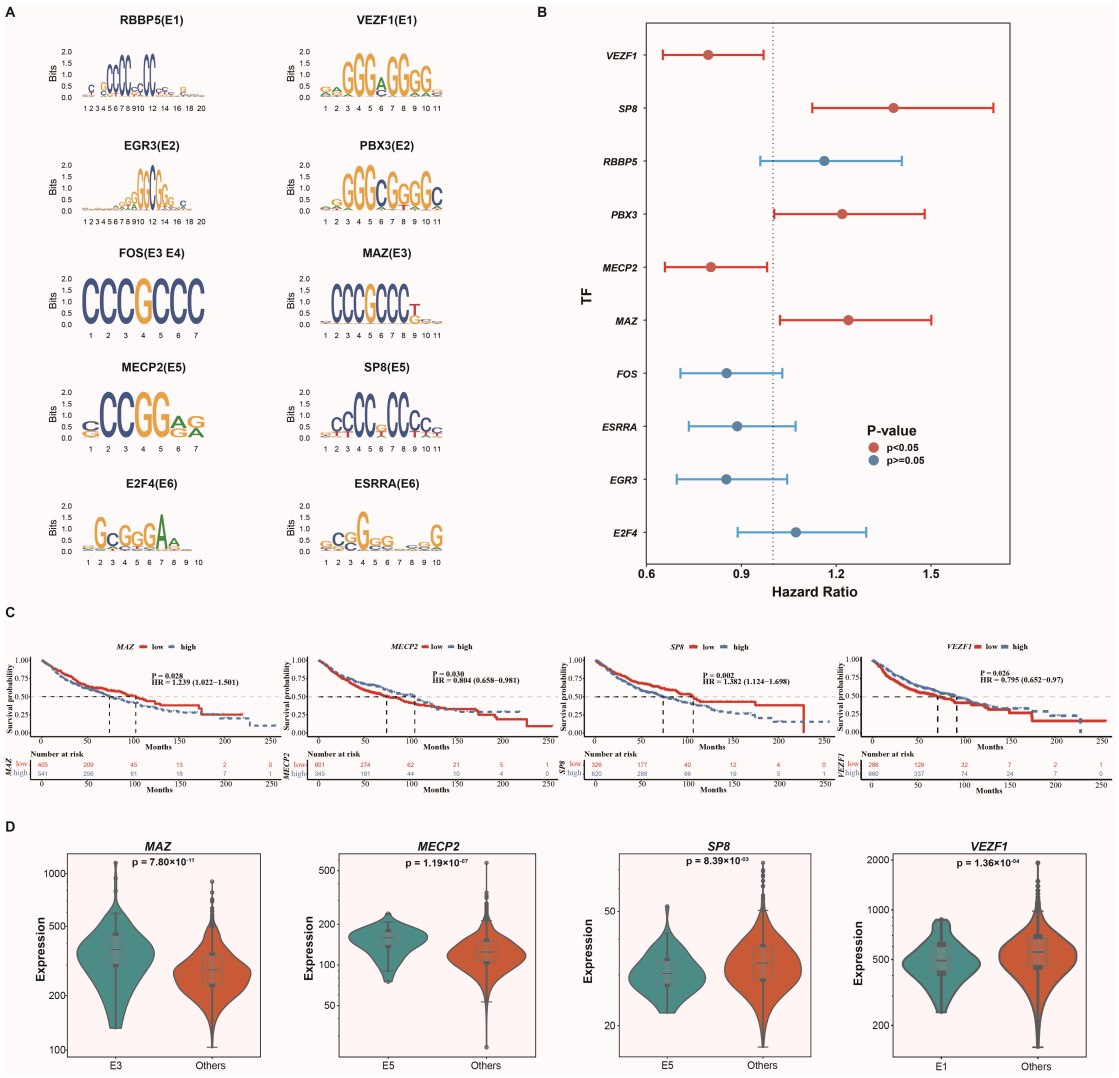
Transcriptional regulatory landscape and prognostic significance of ecotype-associated TFs in the discovery cohort. (**A**) Sequence logos showing the DNA-binding motifs of representative TSs associated with different ecotypes (E1–E6), including RBBP5, VEZF1, EGR3, PBX3, FOS, MAZ, MECP2, SP8, E2F4, and ESRRA. (**B**) Forest plot showing hazard ratios (HRs) and prognostic significance of ecotype-associated TFs in NSCLC patients. Central dots represent HR values, and horizontal lines indicate 95% confidence intervals. (**C**) Kaplan–Meier survival analyses evaluating the prognostic significance of representative TFs, including MAZ, MECP2, SP8, and VEZF1, in the discovery cohort. Patients were stratified into high- and low-expression groups based on transcription factor expression levels, and corresponding log-rank *p*-values are shown. (**D**) Violin plots showing differential expression levels of representative TFs in their corresponding ecotypes relative to other ecotypes in the discovery cohort, including MAZ in E3, MECP2 and SP8 in E5, and VEZF1 in E1. Corresponding *p*-values are indicated in each panel.

Among the identified TFs, FOS emerged as a potential upstream regulator of E3 and E4 ecotypes (Figure 5A). Previous studies have implicated FOS in NSCLC progression, immune regulation, and therapeutic resistance [23]. A FOS-centered transcriptional regulatory network was subsequently constructed using the DoRothEA database with confidence level A (Figure S9). This network identified 88 expressed downstream target genes regulated by FOS, including genes associated with cell proliferation (*CCND1*, *MYC*), apoptosis (*FAS*, *BCL2L11*), inflammatory responses (*IL1B*, *IL6*, *CXCL8*), extracellular matrix remodeling (*MMP1*, *MMP9*), and angiogenesis-related signaling (*VEGFA*, *FGF2*). These findings indicate that FOS may function as a central regulator coordinating multiple oncogenic and immune-related pathways in NSCLC.

GO biological process enrichment analysis further demonstrated that FOS target genes were significantly enriched in inflammatory responses, immune regulation, apoptosis-associated processes, and miRNA regulatory pathways (Figure S10). Collectively, these findings suggest that FOS-regulated genes participate in diverse biological processes related to inflammation, cell survival, stress responses, and post-transcriptional regulation. In addition, MAZ was identified as another potential regulator associated with E3-specific upregulated genes (Figure 5A). Previous studies have reported that elevated *MAZ* expression promotes cell proliferation and immune evasion in lung adenocarcinoma and is associated with poor clinical outcomes [24]. Furthermore, *SP8*, which has previously been implicated in chemoresistance in lung cancer, was predicted to regulate genes highly expressed in E5 (Figure 5A).

The prognostic significance of the top two enriched TFs in each ecotype was further evaluated in the discovery cohort. Univariable Cox regression analysis demonstrated that elevated expression of *SP8*, *PBX3*, and *MAZ* was significantly associated with increased hazard risk, whereas high expression of *VEZF1* and *MECP2* was associated with favorable prognosis (Figure 5B). Kaplan–Meier survival analysis further confirmed significant associations between TF expression and patient survival outcomes (Figure 5C). Specifically, patients with high *MAZ* expression exhibited significantly shorter overall survival than those with low *MAZ* expression (log-rank test, *p*-value = 0.028). Similarly, elevated SP8 expression was associated with unfavorable prognosis (log-rank test, *p*-value = 0.002). In contrast, higher expression levels of *MECP2* (log-rank test, *p*-value = 0.030) and *VEZF1* (log-rank test, *p*-value = 0.026) were associated with prolonged overall survival (Figure 5C).

We next evaluated TF expression patterns across different ecotypes (Figure 5D). *MAZ* expression was significantly elevated in the E3 subgroup compared with all other ecotypes (Wilcoxon test, *p*-value = 7.80 × 10^−11^). In the E5 subgroup, *MECP2* expression was significantly increased (Wilcoxon test, *p*-value = 1.19 × 10^−7^), whereas *SP8* expression was significantly reduced (Wilcoxon test, *p*-value = 8.39 × 10^−3^), consistent with the favorable prognostic characteristics associated with E5. In addition, *VEZF1* expression was significantly decreased in the E1 subgroup (Wilcoxon test, *p*-value = 1.36 × 10^−4^). These findings suggest that distinct TFs may exert ecotype-specific regulatory functions in NSCLC.

### 2.5. Spatial Microenvironment Atlas of Distinct Cell States

**Figure 6:**
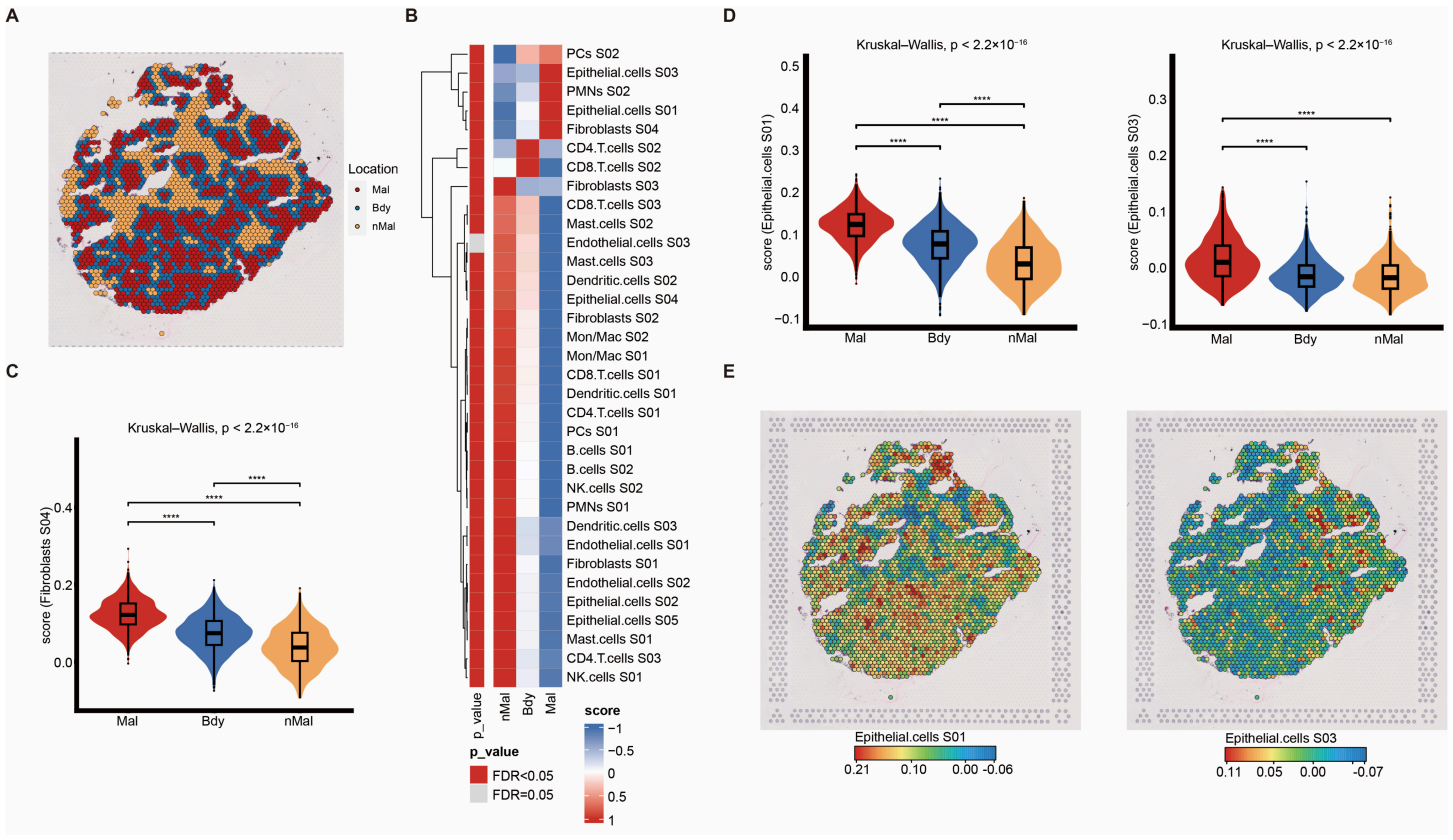
Spatial transcriptomics-based validation of NSCLC-associated cell states. (**A**) NSCLC tissue section annotated with Mal, Bdy, and nMal regions. (**B**) Heatmap showing enrichment scores of transcriptionally defined cell states across Mal, Bdy, and nMal regions. Violin plots showing spatial enrichment scores of representative cell states, including fibroblast state S04 (**C**) and epithelial cell states S01 and S03 (**D**), across Mal, Bdy, and nMal regions. Statistical significance was assessed using the Kruskal–Wallis test, and corresponding *p*-values are shown. (**E**) Spatial enrichment analysis showing differential localization patterns of epithelial cell states S01 and S03 within NSCLC tissue regions. Violin plots show the distributions of cell state scores across 966 Mal, 816 Bdy, and 587 nMal spots from the representative P19_T2 Visium tissue section of patient 19. The corresponding Kruskal–Wallis ε^2^ effect sizes for fibroblasts S04, epithelial cells S01, and epithelial cells S03 are 0.389, 0.381, and 0.127, respectively. These spot-level analyses are exploratory and are intended to illustrate spatial heterogeneity within the tissue section rather than to provide patient-level statistical inference. In this figure, **** indicates a *p*-value < 2.20 × 10^−16^.

To further characterize the spatial microenvironment associated with distinct cell states in NSCLC, we applied the Cottrazm framework [25] to integrate spatial transcriptomics, single-cell transcriptomics, and H&E-stained histological images. This approach enabled high-resolution reconstruction of tumor boundary microenvironments and spatial characterization of cell-state distributions across tissue sections. The spatial transcriptomic analysis was performed using one representative Visium tissue section (P19_T2) from patient 19, comprising 2369 spots, including 966 malignant (Mal) region spots, 816 boundary (Bdy) region spots, and 587 non-malignant (nMal) region spots. Regional differences were assessed using the Kruskal–Wallis test, and the corresponding ε^2^ statistic was reported as a measure of effect size. Because all spots originated from the same tissue section, the spot-level analyses were considered exploratory and were not used for patient-level statistical inference. Spatial transcriptomics datasets were processed to identify Mal, Bdy, and nMal regions based on spatial transcriptomic profiles, tissue morphology, and inferred copy number variation patterns (Figure 6A). Subsequently, enrichment scores for the 34 cell states identified by EcoTyper were calculated across Mal, Bdy, and nMal regions in the 10x Genomics Visium spatial transcriptomics validation cohort.

Global comparison of cell state enrichment scores revealed significant spatial heterogeneity in 33 of the 34 identified cell states across the three spatial regions (Figure 6B). Notably, fibroblast state S04 was significantly enriched in Mal regions compared with Bdy and nMal regions (Kruskal–Wallis test, *p*-value < 2.20 × 10^−16^), suggesting its potential involvement in tumor-associated stromal remodeling and malignant progression (Figure 6C). Spatial mapping further demonstrated that epithelial cell states S01 and S03 were predominantly localized within Mal regions relative to Bdy and nMal regions (Figure 6D,E; Kruskal–Wallis test, *p* value < 2.20 × 10^−16^). These findings highlight the pronounced spatial heterogeneity of cell states within the NSCLC tumor microenvironment and suggest distinct spatial niches associated with tumor progression.

## 3. Discussion

High-resolution characterization of the TME is essential for understanding NSCLC progression, identifying clinically relevant tumor ecosystems, and developing more effective precision therapeutic strategies [26,27,28]. Given the substantial cell and molecular heterogeneity of NSCLC, systematic delineation of multicellular programs within the TME may provide important insights into tumor progression, immune regulation, and therapeutic responses [29,30,31,32,33]. In this study, we applied the EcoTyper framework to bulk transcriptomic datasets from NSCLC patients and identified 34 transcriptionally distinct cell states across 12 major cell types, together with six recurrent multicellular ecotypes. These cell states and ecotypes were reproducibly validated across multiple independent bulk transcriptomic cohorts, as well as scRNA-seq and spatial transcriptomics cohorts, highlighting the robustness and cross-platform reproducibility of the identified multicellular landscape. Collectively, these findings provide a comprehensive atlas of NSCLC-associated cell states and multicellular ecosystems and further emphasize the extensive heterogeneity of the NSCLC microenvironment.

We further demonstrated that a substantial proportion of the identified cell states and ecotypes were significantly associated with patient prognosis. Notably, several immune-associated cell states, including CD8+ T cell state S03 and dendritic cell state S03, were associated with favorable prognosis, whereas fibroblast and epithelial cell states were linked to poor prognosis. These findings are consistent with previous studies demonstrating that immune activation is generally associated with improved survival, whereas stromal remodeling and malignant epithelial programs contribute to tumor progression and unfavorable outcomes in NSCLC. In addition, the prognostic model constructed using cell state marker genes exhibited stable predictive performance across independent cohorts, suggesting potential utility for risk stratification and prognostic assessment in NSCLC patients.

Functional enrichment analyses further demonstrate substantial functional diversity among distinct cell states. Multiple states were enriched in immune-related pathways, inflammatory signaling, and cytokine-associated programs, indicating extensive immune and stromal heterogeneity within the NSCLC microenvironment. Moreover, TF enrichment analysis identified several ecotype-associated transcriptional regulators, including FOS, MAZ, and SP8. Previous studies have implicated these TFs in tumor proliferation, immune regulation, and therapeutic resistance in lung cancer. Our findings further suggest that ecotype-specific transcriptional programs may contribute to shaping multicellular ecosystem architecture and influencing clinical outcomes in NSCLC.

Spatial transcriptomics analysis further revealed pronounced spatial heterogeneity of cell states within tumor tissues. Most cell states displayed significantly different enrichment patterns across Mal, Bdy, and nMal regions. In particular, fibroblast state S04 was predominantly enriched in Mal regions, suggesting its potential involvement in tumor-associated stromal remodeling and invasive microenvironment formation. These findings highlight the existence of distinct spatial ecological niches within the NSCLC microenvironment and underscore the importance of integrating spatial context into ecosystem-level analyses. In addition, we observed significant associations between cell states and predicted therapeutic sensitivity profiles. Multiple cell states were correlated with sensitivity to chemotherapeutic and targeted agents, including cisplatin, trametinib, and afatinib. These findings suggest that cell state-based stratification may provide clinically relevant information for therapeutic selection and precision treatment optimization in NSCLC.

The batch effects among seven NSCLC cohorts were corrected using the ComBat algorithm (Figure S11). As illustrated in Figure S11, samples from different cohorts became well intermingled, and the dominant batch-related variation was effectively removed. We fully acknowledge that complete elimination of batch effects is rarely achievable, and some residual technical variation may remain. However, the marked reduction in PCs and the improved mixing across datasets in PCA indicate that the correction was sufficient for reliable downstream differential expression and classification analyses. A total of 674 lung adenocarcinoma (LUAD) samples and 272 lung squamous cell carcinoma (LUSC) samples were collected from seven GPL570 cohorts and used to construct the LUAD and LUSC discovery cohorts, respectively. Batch effects among gene expression profiles within the LUAD discovery cohort and the LUSC discovery cohort were independently corrected using the ComBat algorithm (Figures S12 and S13). Subsequently, the EcoTyper framework was applied separately to identify cell states and ecotypes in the LUAD and LUSC discovery cohorts.

In the LUAD discovery cohort, 34 distinct cell states across 12 major cell types were identified by EcoTyper, with each cell type containing 2–4 transcriptionally distinct states (Figure S14A). Distinct distribution patterns of the inferred cell states were observed within each cell type (Figure S14B). To further characterize the interactions among different cell states, we employed EcoTyper to delineate coordinated multicellular ecosystems. This analysis revealed three distinct ecotypes in LUAD, each comprising 7–14 interacting cell states that consistently co-occurred within the TME (Figure S14C,D). Similarly, EcoTyper delineated 31 distinct cell states and four multicellular ecotypes within the LUSC discovery cohort, with each ecotype consisting of 4–15 coexisting and interacting cell states (Figure S15). Together, these findings provide a comprehensive characterization of multicellular ecotypes in LUAD and LUSC, enhancing our understanding of the distinct cellular organization patterns and complex interactions within the TME of these two major lung cancer subtypes.

Nevertheless, several limitations of this study should be acknowledged. First, because of incomplete clinical and pathological annotations in several discovery and validation cohorts, our analyses primarily focused on prognostic associations. Additional investigations incorporating detailed clinicopathological characteristics, treatment information, and molecular subtypes are warranted in future studies. Second, owing to the lack of NSCLC cohorts containing both comprehensive treatment response and survival data, therapeutic sensitivity was estimated using the gene expression-based prediction framework oncoPredict rather than real-world clinical response data. Therefore, these findings should be interpreted cautiously and require further validation in independent clinical cohorts with matched treatment and outcome information.

## 4. Materials and Methods

### 4.1. Data Source

Bulk RNA-seq data and corresponding clinical information of NSCLC patients were downloaded from The Cancer Genome Atlas (TCGA) and Gene Expression Omnibus (GEO) databases. Only patients with LUAD and LUSC from these databases were included in this study. In total, 3486 NSCLC patients from 18 public cohorts were enrolled, including 516 patients from the TCGA-LUAD cohort, 501 patients from the TCGA-LUSC cohort, 443 patients from the GSE68465 cohort, and 199 patients from the GSE81089 cohort. For NSCLC GEO cohorts with raw CEL files generated using Affymetrix platforms, the robust multi-array average (RMA) algorithm implemented in the R package affy (version 1.44.0) was applied for data preprocessing [34]. Probes matching more than one gene were excluded. For genes corresponding to multiple probes, the probe with the highest average expression level was selected to represent the expression value of the gene. Detailed information for all cohorts is provided in Table S2.

Seven cohorts from the GPL570 platform (GSE30219, GSE31210, GSE50081, GSE19188, GSE3141, GSE37745, and GSE29013), comprising 946 NSCLC patients with complete overall survival information, were integrated to construct the discovery cohort. Batch effects among the gene expression profiles were corrected using the ComBat algorithm implemented in the R package sva (version 3.52.0) [35]. The remaining 11 NSCLC cohorts, including TCGA-LUAD and TCGA-LUSC, served as validation cohorts. Additionally, 10x Genomics Visium spatial transcriptomics data from NSCLC patients, obtained from the ArrayExpress database under accession number E-MTAB-13530 [36], were further used as an independent validation cohort.

For the GEO datasets GSE30219, GSE31210, GSE50081, GSE19188, GSE37745, GSE29013, GSE10245, GSE14814, GSE31547, GSE68465, GSE41271, and GSE13213, raw CEL files were available and were processed using the RMA algorithm. Because RMA outputs log2-transformed expression values, the resulting expression matrices were converted back to the original linear scale using the inverse transformation (2^x) before EcoTyper analysis. For GSE8894, only log2-transformed expression data were available from the GEO repository and were similarly back-transformed to the linear scale using 2^x. In contrast, the expression matrices for GSE3141, GSE4573, and GSE81089 were not log2-transformed and were therefore used directly. For the TCGA-LUAD and TCGA-LUSC cohorts, FPKM-normalized RNA-sequencing expression profiles were used as EcoTyper input.

The NSCLC single-cell dataset GSE131907 [37], comprising 58 lung adenocarcinoma samples from 44 patients, was retrieved from the GEO database. Raw count matrices for each sample were converted into Seurat objects, and subsequent analyses were conducted using the Seurat package (version 5.4.0) [38]. During quality control, cells with fewer than 500 or more than 6000 detected genes, fewer than 500 unique molecular identifier (UMI) counts, or mitochondrial gene expression exceeding 10% were excluded. Raw UMI counts were subsequently normalized using the NormalizeData function. Cell cycle effects were estimated by calculating S and G2M scores with the CellCycleScoring function and regressed out during data scaling using the ScaleData function. Subsequently, the top 2000 highly variable genes were identified using the FindVariableFeatures function for downstream analysis.

### 4.2. EcoTyper Framework

In this study, the cell states and ecotypes of lung cancer patients were identified using the EcoTyper framework (https://ecotyper.stanford.edu/, accessed on 15 June 2026) [15]. First, cell-type fractions were inferred from bulk transcriptomic profiles of each sample using CIBERSORTx (https://cibersortx.stanford.edu/, accessed on 15 June 2026) with default parameters and two validated signature matrices, TR4 and LM22 [39]. Based on the inferred cell-type-specific expression profiles, EcoTyper was applied to identify distinct cell states within each major cell type using non-negative matrix factorization (NMF) across multiple factorization ranks. The optimal rank was selected based on cophenetic coefficient stability according to the recommended heuristic criterion. To ensure the identification of high-confidence cellular states, EcoTyper incorporates two quality control procedures, including adaptive false-positive index (AFI)-based filtering to remove false-positive states and exclusion of cell states containing fewer than 10 marker genes.

Following quality control, EcoTyper identified recurrent co-occurrence patterns among cell states across samples to define ecotypes within the discovery cohort. Pairwise overlap among cell states was quantified using the Jaccard index with statistical filtering, followed by hierarchical clustering and silhouette width optimization. Ecotypes containing fewer than three cell states were excluded from subsequent analyses.

In addition, the EcoTyper framework was applied to recover previously defined cell states and associated ecotypes from external validation cohorts. For each cell type, EcoTyper derived cell state-specific expression signatures from the discovery cohort and projected them onto external cohorts. Recovery of cell states in the validation cohorts was assessed using permutation testing, and statistical significance was determined based on z-scores. Cell states with z-scores > 1.65 and one-sided *p*-values < 0.05 were considered significantly recovered.

### 4.3. Transcriptional Regulatory Analysis of Ecotypes

To identify potential transcriptional regulators associated with NSCLC ecotypes, significantly upregulated genes in each ecotype were first identified using the Wilcoxon rank-sum test. Genes with adjusted *p*-values < 0.05 were subjected to transcription factor (TF) binding motif enrichment analysis using the RcisTarget package (version 1.16.0) and the “hg19-500bp-upstream-7species.mc9nr.genes_vs_motifs.rankings.feather” database. TF motif enrichment analysis was performed using the cisTarget function with a normalized enrichment score (NES) threshold of 2.5. The AUC threshold was determined as the mean AUC plus 2.7 × SD (AUC).

### 4.4. Construction and Validation of the Risk Score Model

A total of 823 marker genes associated with cell states identified in the discovery cohort were used to construct and validate a prognostic risk score model for NSCLC patients. First, univariate Cox proportional hazards regression analysis was performed using the R package survival to identify marker genes significantly associated with overall survival in the discovery cohort (*p*-value < 0.05). A total of 111 prognostic genes were subsequently subjected to LASSO Cox regression analysis using the glmnet package (version 4.1-10). Based on the optimal penalty parameter (lambda.min), 42 genes were retained for model construction. Risk scores for individual patients were calculated using the coefficients derived from the LASSO Cox regression model. Patients in the discovery cohort were subsequently stratified into high- and low-risk groups according to the median risk score. The predictive performance of the risk score model was further validated in the TCGA cohort.

### 4.5. Statistical Analysis

Functional enrichment analysis of cell state marker genes was performed using the R package clusterProfiler (version 4.14.6) [40]. Gene Ontology (GO) biological process gene sets, Hallmark gene sets, and immune-related gene sets were used for enrichment analysis. Hallmark gene sets were obtained from the Molecular Signatures Database (MSigDB) [41], while immune-related gene sets were curated from the ImmPort database [42].

Univariate Cox regression, multivariable Cox regression and survival analyses were conducted using the R package survival (version 3.8-6). Drug response in lung cancer patients from the TCGA cohort was predicted using the R package oncoPredict (version 1.2) [43]. Data visualization, including UMAP, bar plots, survival curves, and violin plots, was performed using the Python package Matplotlib (version 3.10.8). Circos plots were generated using the R package ggplot2 (version 4.0.1). Heatmaps were generated using Matplotlib or the R package pheatmap (version 1.0.13). All statistical analyses were performed using R software (version 4.4.0) and Python (version 3.12). To correct for multiple comparisons, we applied the Benjamini–Hochberg (BH) false discovery rate (FDR) procedure. All statistical tests were two-sided, and *p*-values < 0.05 or FDR-adjusted q-values < 0.05 were considered statistically significant.

## 5. Conclusions

In summary, this study provides a systematic in silico characterization of the multicellular ecosystem landscape in NSCLC using the EcoTyper framework. We delineated 34 transcriptionally distinct cell states and six recurrent ecotypes, which were consistently observed across bulk transcriptomic, single-cell, and spatial transcriptomic datasets. Our analyses revealed that these multicellular programs are potentially associated with patient prognosis, biological functions, spatial organization, and predicted therapeutic sensitivity, suggesting their possible clinical relevance. Collectively, these computational findings contribute to the current understanding of NSCLC TME heterogeneity and illustrate the utility of ecosystem-level analyses for generating biologically and clinically meaningful hypotheses. However, we acknowledge that all results are derived purely from bioinformatics predictions and lack experimental corroboration. Therefore, while this work provides a preliminary computational atlas and a reference point for future studies, the proposed prognostic biomarkers and therapeutic predictions should be interpreted with caution and require rigorous experimental validation, as well as prospective clinical evaluation, before any translational application can be considered.

## Data Availability

The datasets supporting the conclusions of this article are available in the GEO (http://www.ncbi.nlm.nih.gov/geo/, accessed on 15 June 2025), TCGA (https://portal.gdc.cancer.gov/, accessed on 10 July 2025) and ArrayExpress (https://www.ebi.ac.uk/biostudies/arrayexpress, accessed on 21 July 2025) databases. A list of accessions of publicly available datasets used in this study can also be found in Table S2. The source code used in this study is available at the following GitHub repository: https://github.com/LeiyangHarbin/NSCLC-Ecotype (accessed on 21 July 2025).

## Supplementary Materials

The following supporting information can be downloaded at: https://www.mdpi.com/article/10.3390/ijms27156718/s1.

## Abbreviations

NSCLC	Non-small cell lung cancer
TME	Tumor microenvironment
TCGA	The Cancer Genome Atlas
GEO	Gene Expression Omnibus
LUAD	Lung adenocarcinoma
LUSC	Lung squamous cell carcinoma
RMA	Robust multi-array average
ADC	Adenocarcinoma
SCC	Squamous cell carcinoma
EMT	Epithelial–mesenchymal transition
HR	Hazard ratios
TF	Transcription factor
NMF	Non-negative matrix factorization
AFI	Adaptive false-positive index
NES	Normalized enrichment score
MSigDB	Molecular Signatures Database
GO	Gene Ontology
