# Supplementary material for: Landscape of Cell States and Multicellular Ecotypes in Non-Small Cell Lung Cancer
Source: Int J Mol Sci. 2026 Jul 28;27(15):6718. doi: 10.3390/ijms27156718 (PMC13467122; doi:10.3390/ijms27156718)
Supplement: Supplementary file 1 [file ijms-27-06718-s001.zip › Supplementary Figures.pdf]

Supplementary Figure

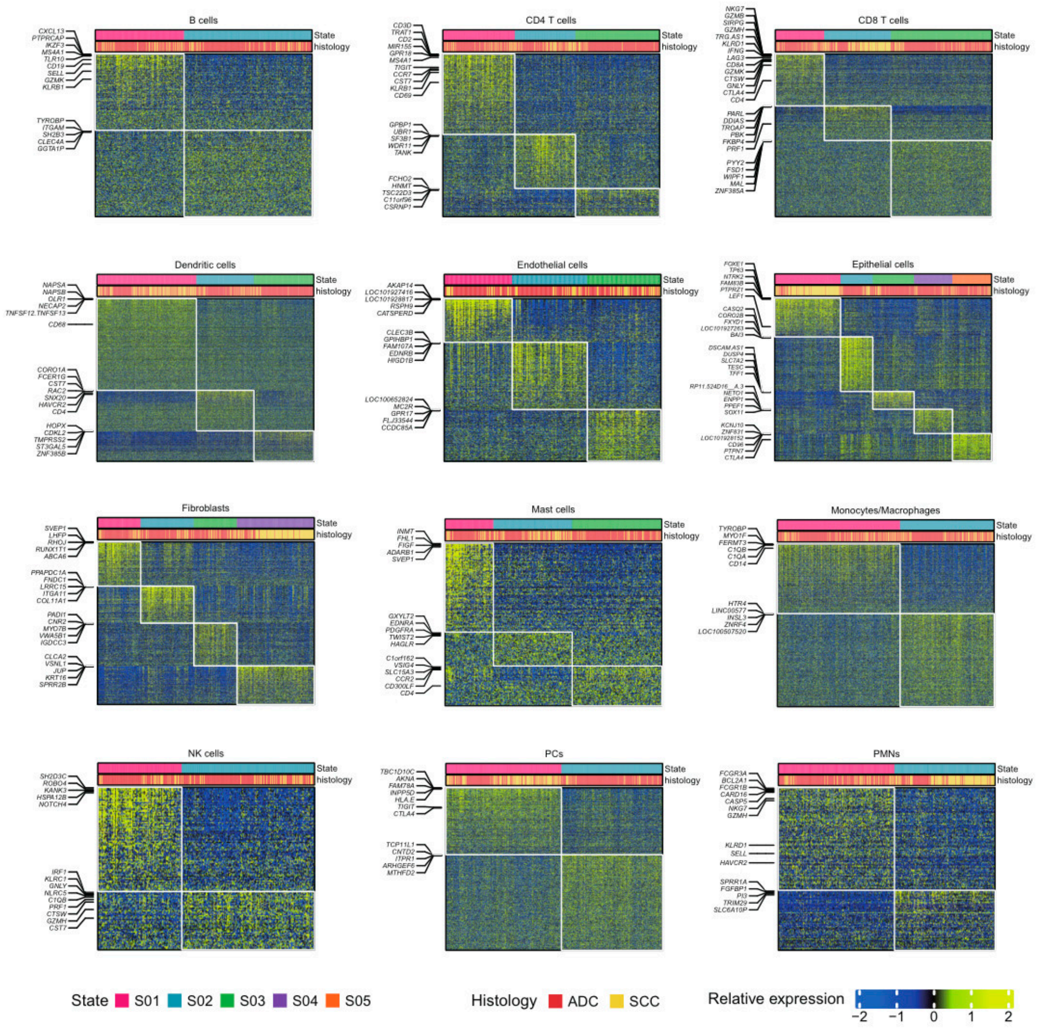

Figure S1. Transcriptionally defined cell states in the discovery cohort.

Heatmap showing transcriptionally defined cell states identified in the discovery cohort together with the corresponding marker genes used for state classification.

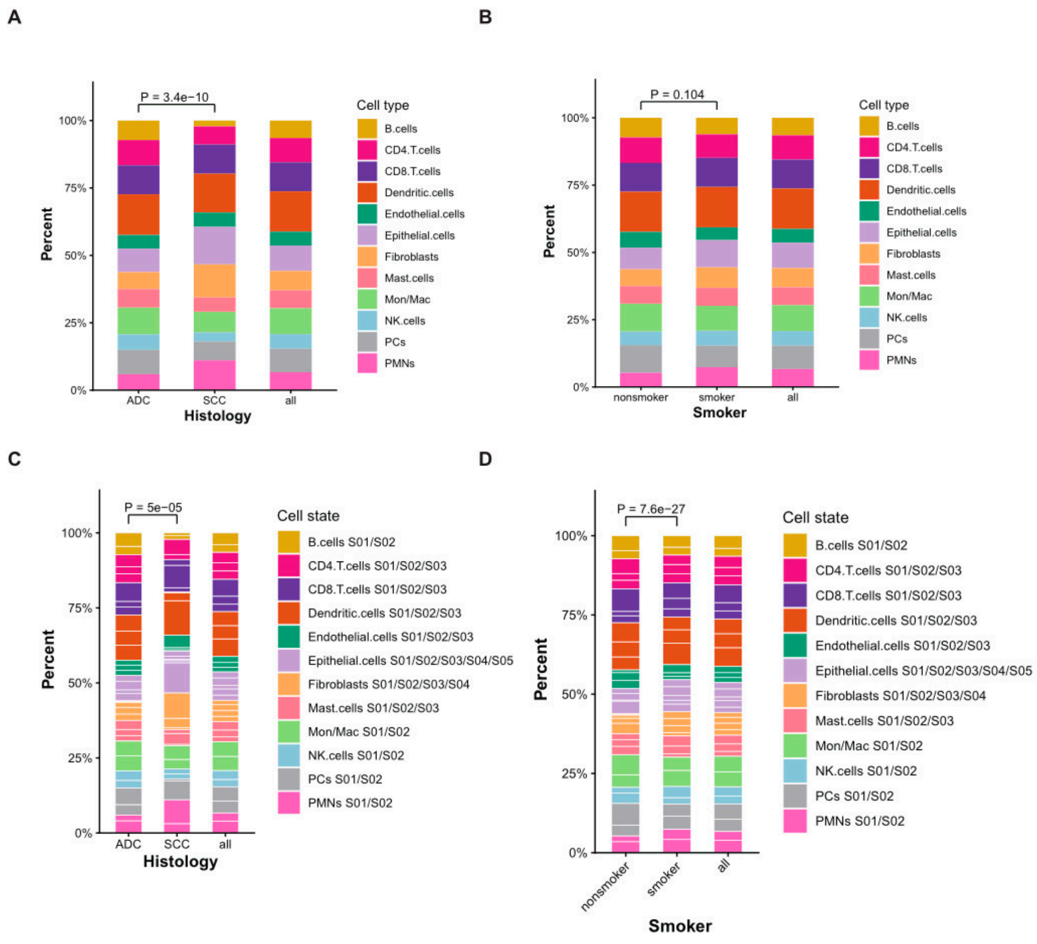

Figure S2. Distribution of cell types and transcriptionally defined cell states according to histological subtype and smoking status in the NSCLC discovery cohort.

(A) Stacked bar plot showing the relative proportions of 12 cell types across different NSCLC histological subtypes, including adenocarcinoma (ADC), squamous cell carcinoma (SCC), and the overall cohort.

(B) Stacked bar plot showing the relative proportions of 12 cell types stratified by smoking status, including non-smokers, smokers, and the overall cohort.

(C) Stacked bar plot showing the relative proportions of 34 cell states across different NSCLC histological subtypes, including ADC, SCC, and the overall cohort.

(D) Stacked bar plot showing the relative proportions of 34 cell states stratified by smoking status,

including non-smokers, smokers, and the overall cohort.

Statistical significance was assessed using the chi-square test, and corresponding P-values are indicated.

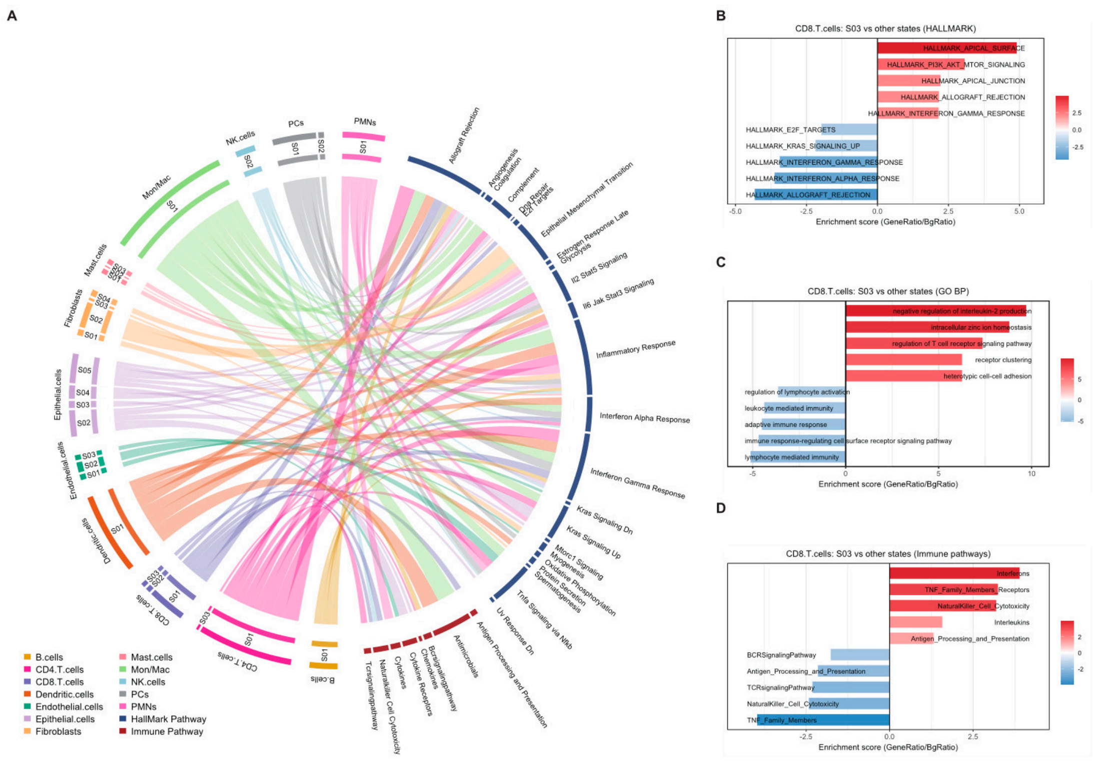

Figure S3. Functional enrichment analysis of cell states in NSCLC.

(A) Circos plot showing enrichment of cell state marker genes in Hallmark pathways and immune-related pathways.

(B-D) Bar plots showing enriched pathways associated with CD8+ T cell state S03 and other cell states. Pathways associated with CD8+ T cell state S03 are highlighted in red, whereas pathways associated with other states are shown in blue. **(B)** Hallmark pathways; **(C)** GO biological processes (GO BP); **(D)** immune-related pathways.

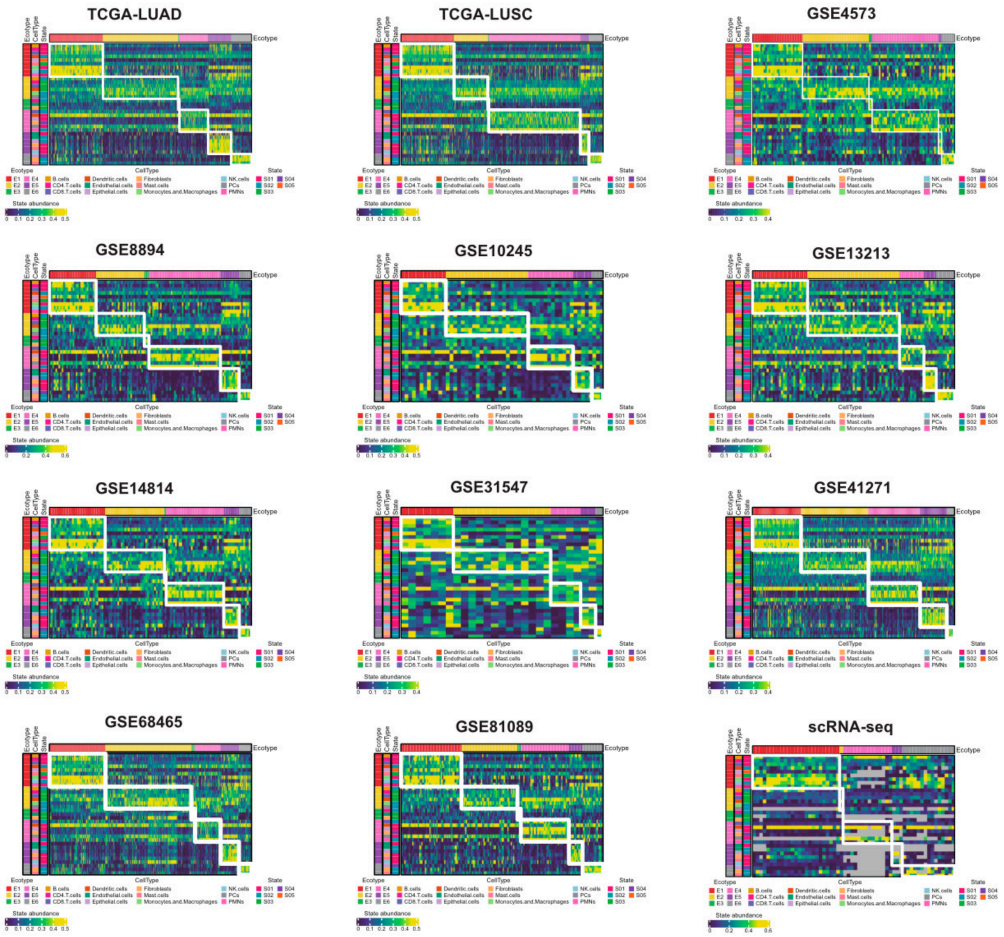

Figure S4. Validation of NSCLC ecotypes across independent cohorts.

Heatmaps showing abundance patterns of cell states grouped according to six ecotypes across independent validation cohorts.

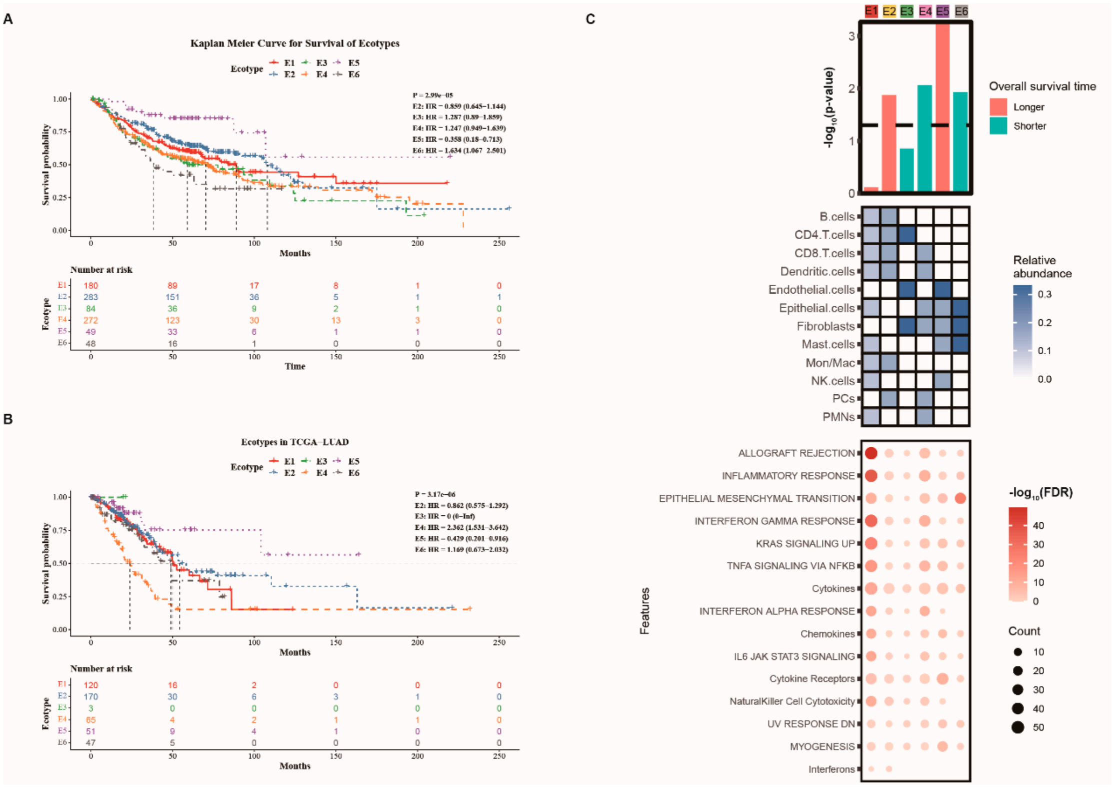

Figure S5. Prognostic and biological characteristics of NSCLC ecotypes.

(A) Kaplan-Meier survival analysis of six ecotypes in the NSCLC discovery cohort.

(B) Kaplan-Meier survival analysis of six ecotypes in the TCGA-LUAD validation cohort, demonstrating the prognostic relevance of ecotype classification in an independent cohort.

(C) Molecular characteristics of six ecotypes. Top panel: associations between ecotypes and patient prognosis; middle panel: abundance of each cell type within ecotypes; bottom panel: functional pathways enriched in different ecotypes.

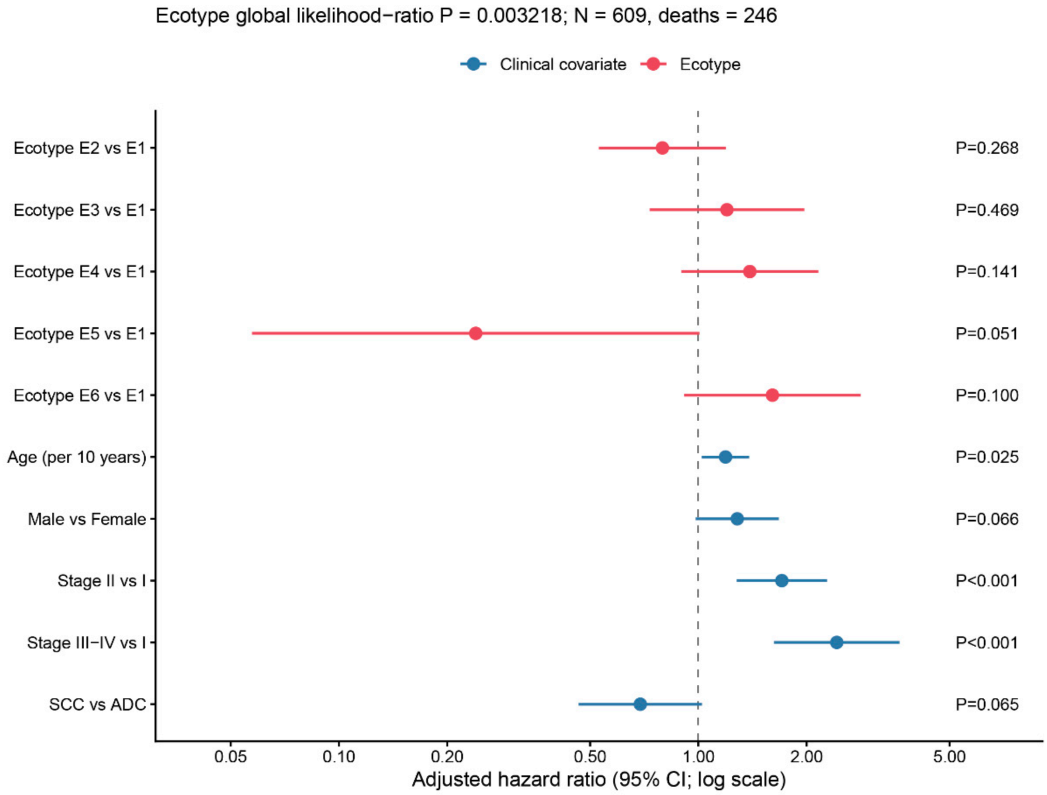

Figure S6. A forest plot of multivariate Cox regression analysis for the NSCLC discovery cohort.

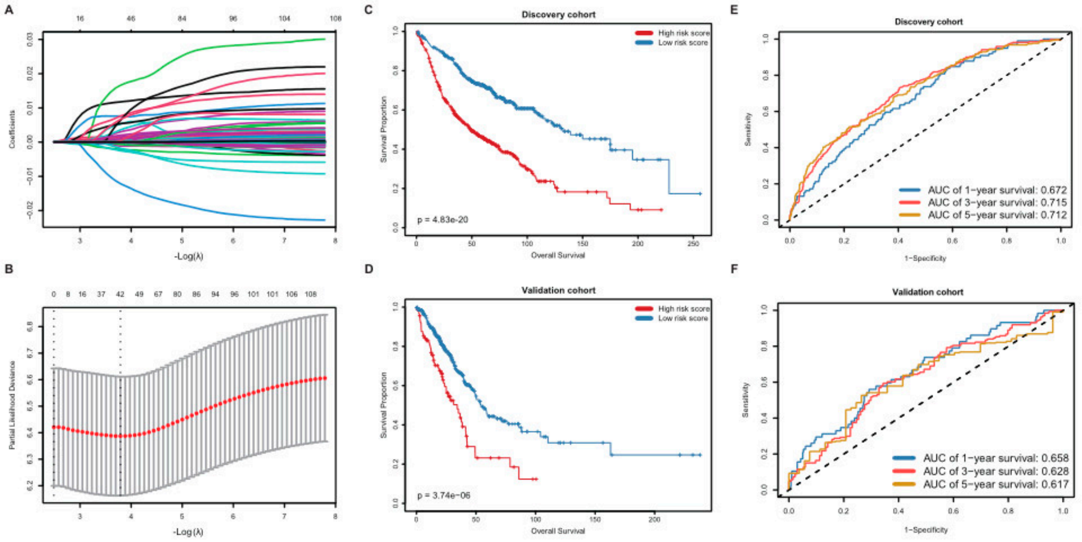

Figure S7. LASSO Cox regression model for prognostic signature selection and survival prediction in discovery and validation cohorts.

(A) LASSO coefficient profiles of candidate prognostic genes.

(B) Partial likelihood deviance plotted against log(λ). The optimal λ value corresponding to the minimum deviance is indicated.

(C, D) Kaplan–Meier survival curves comparing overall survival between high- and low-risk groups in the discovery cohort **(C)** and validation cohort **(D)**.

(E, F) Time-dependent ROC curves for predicting 1-, 3-, and 5-year survival in the discovery cohort **(E)** and validation cohort **(F)**, with corresponding AUC values indicated.

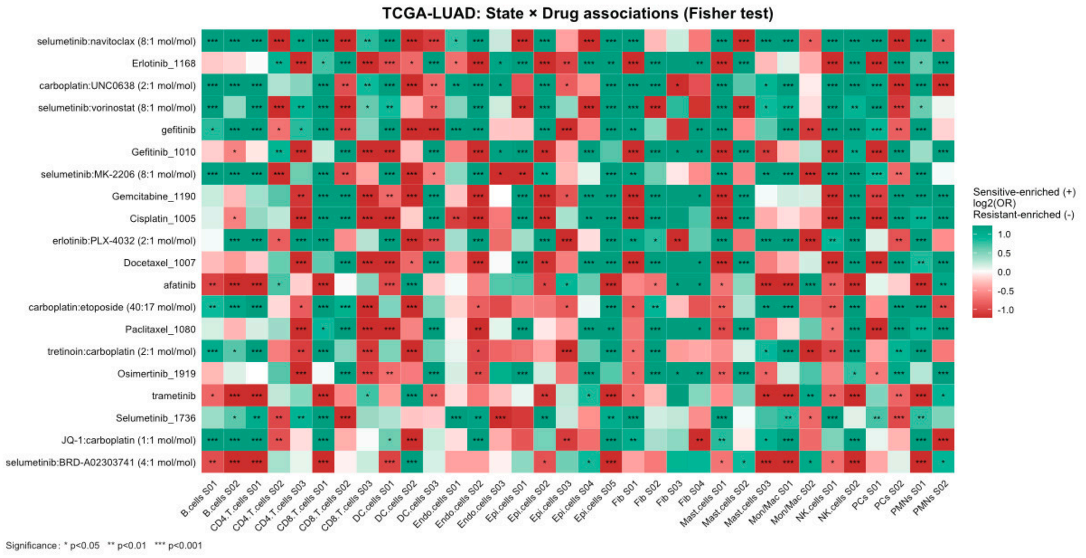

Figure S8. Prediction of drug response in NSCLC based on cell states.

Heatmap showing associations between cell states and predicted responses to therapeutic agents in the TCGA-LUAD cohort.

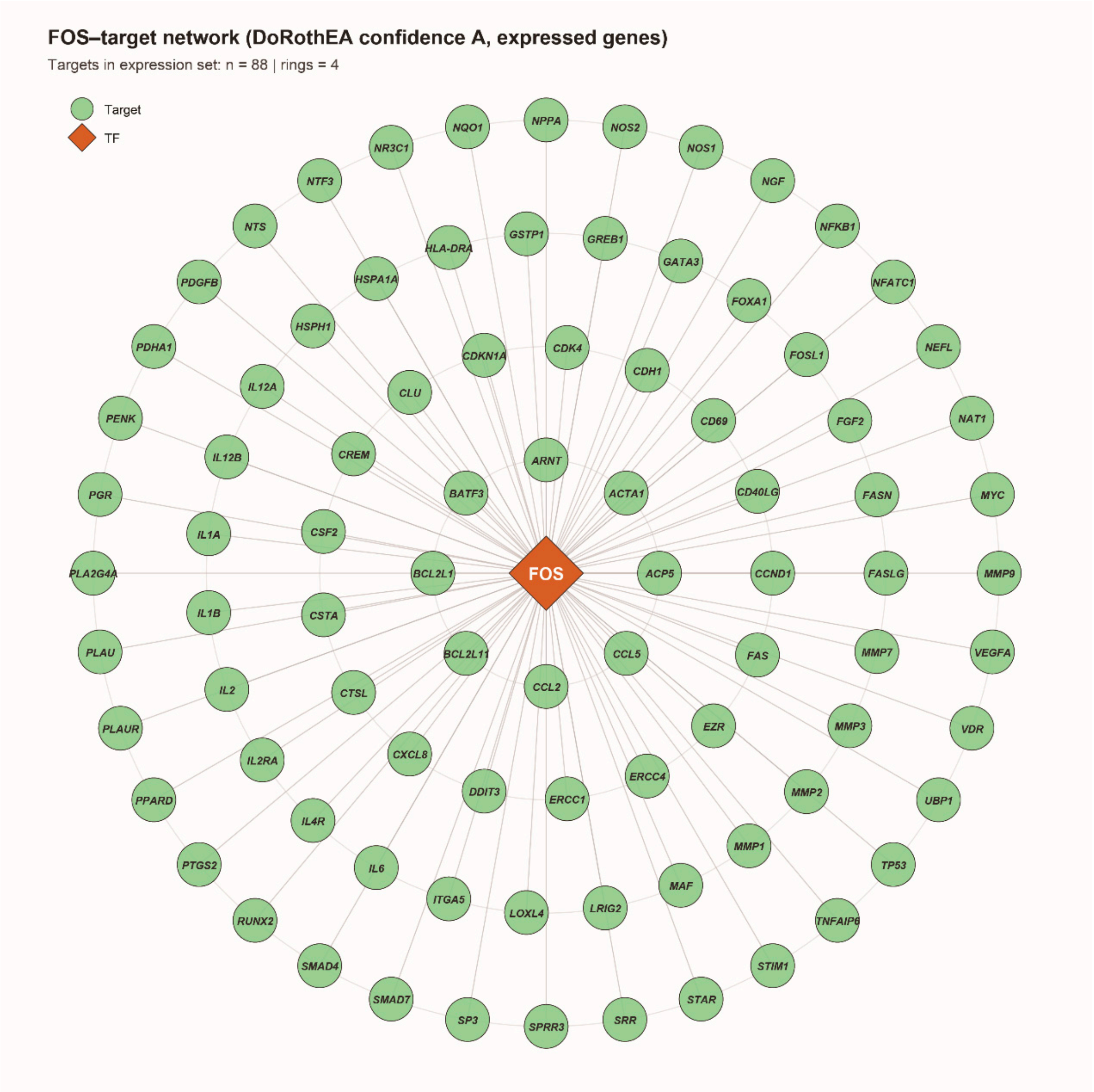

Figure S9. Transcriptional regulatory network of FOS in NSCLC.

Network visualization showing regulatory relationships between FOS and its target genes inferred from the DoRothEA database with confidence level A. The network contains 88 target genes organized into four concentric rings. Nodes represent FOS or its target genes, and edges indicate regulatory interactions.

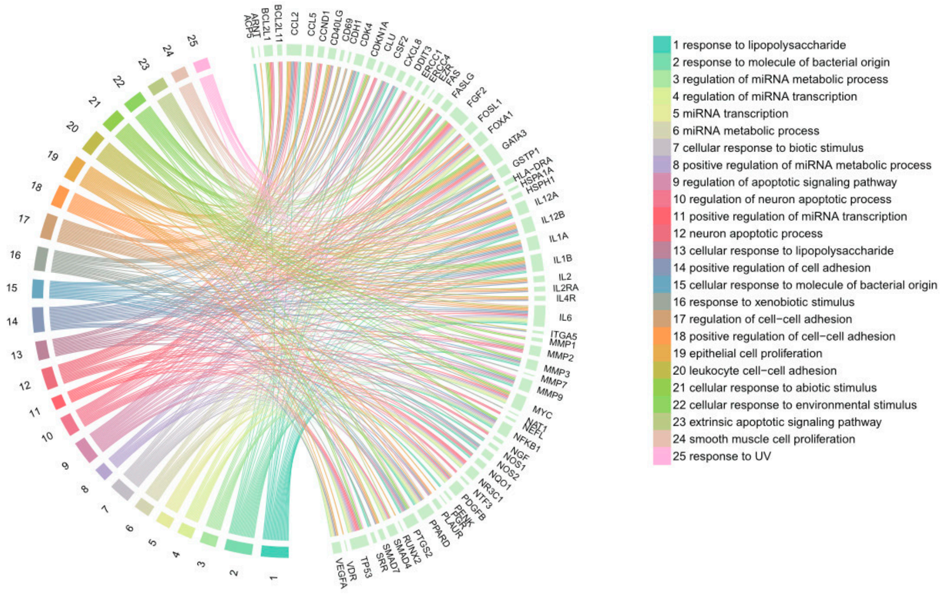

Figure S10. Functional enrichment analysis of transcription factor targets in NSCLC.

Chord diagram showing biological pathways enriched among FOS target genes.

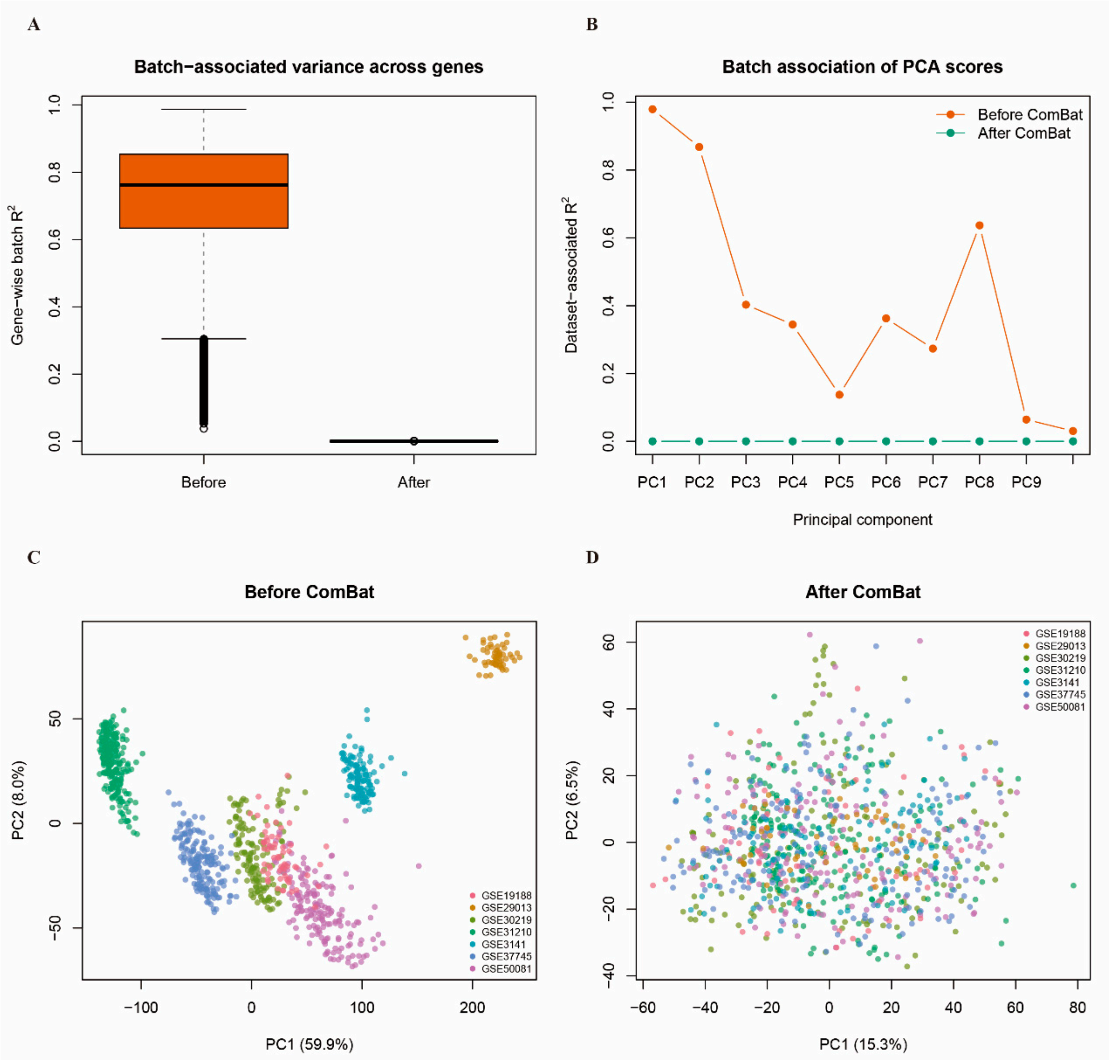

Figure S11. Evaluation of ComBat batch effect correction in discovery cohort.

(A) Gene-wise batch-associated variance (R^2) before and after ComBat correction. **(B)** Dataset-associated variance (R^2) across the first nine principal components before and after correction. PCA plots of the merged GEO datasets before (**C**) and after (**D**) ComBat correction, illustrating effective removal of batch effects while maintaining the global expression structure.

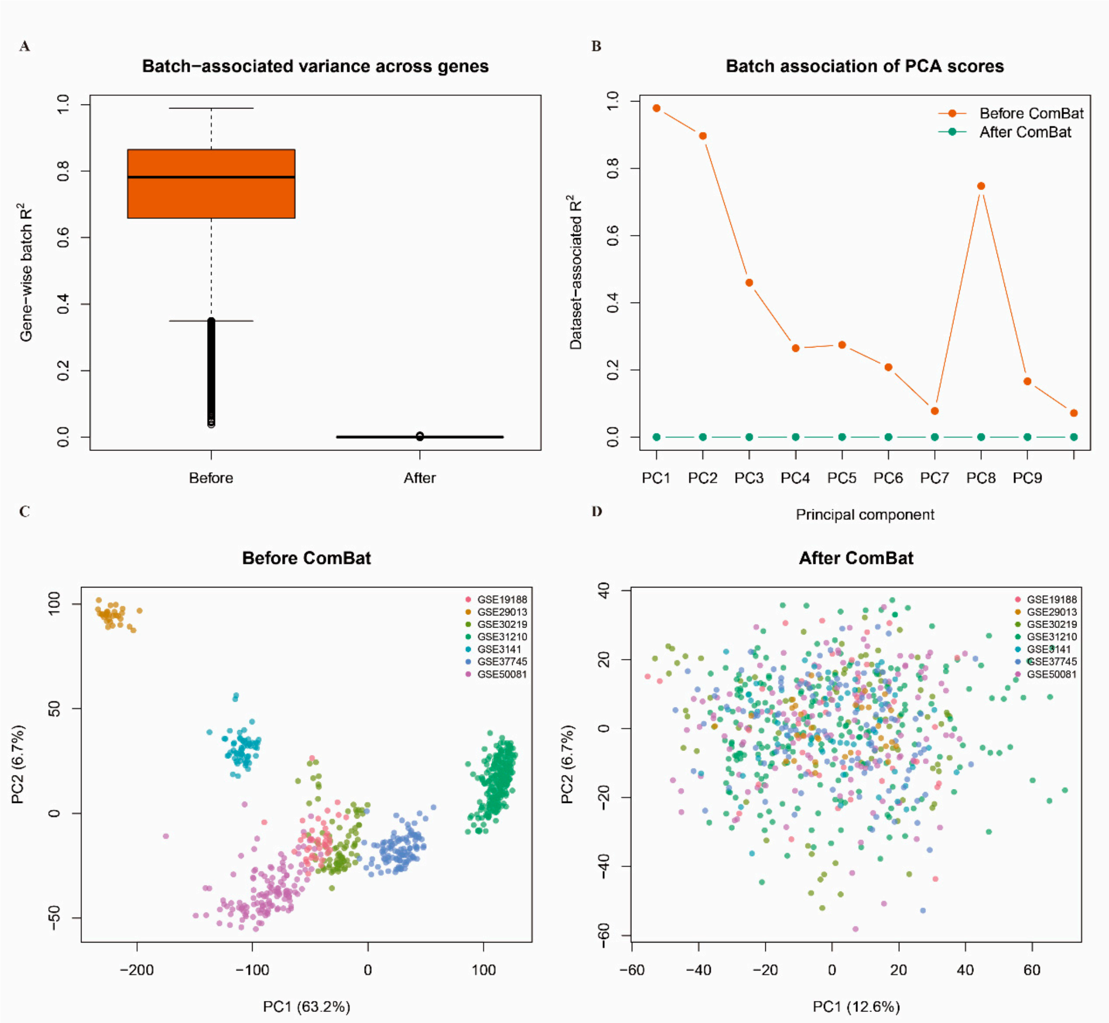

Figure S12. Evaluation of batch effect correction using ComBat in the LUAD cohort.

(A) Distribution of gene-wise batch-associated variance (R^2) before and after ComBat correction. **(B)** Dataset-associated variance (R^2) of the first nine principal components before and after ComBat correction. **(C)** PCA of the merged LUAD cohorts before batch correction, showing clustering of samples by dataset. **(D)** PCA after ComBat correction, demonstrating improved integration of samples across datasets with substantially reduced batch effects.

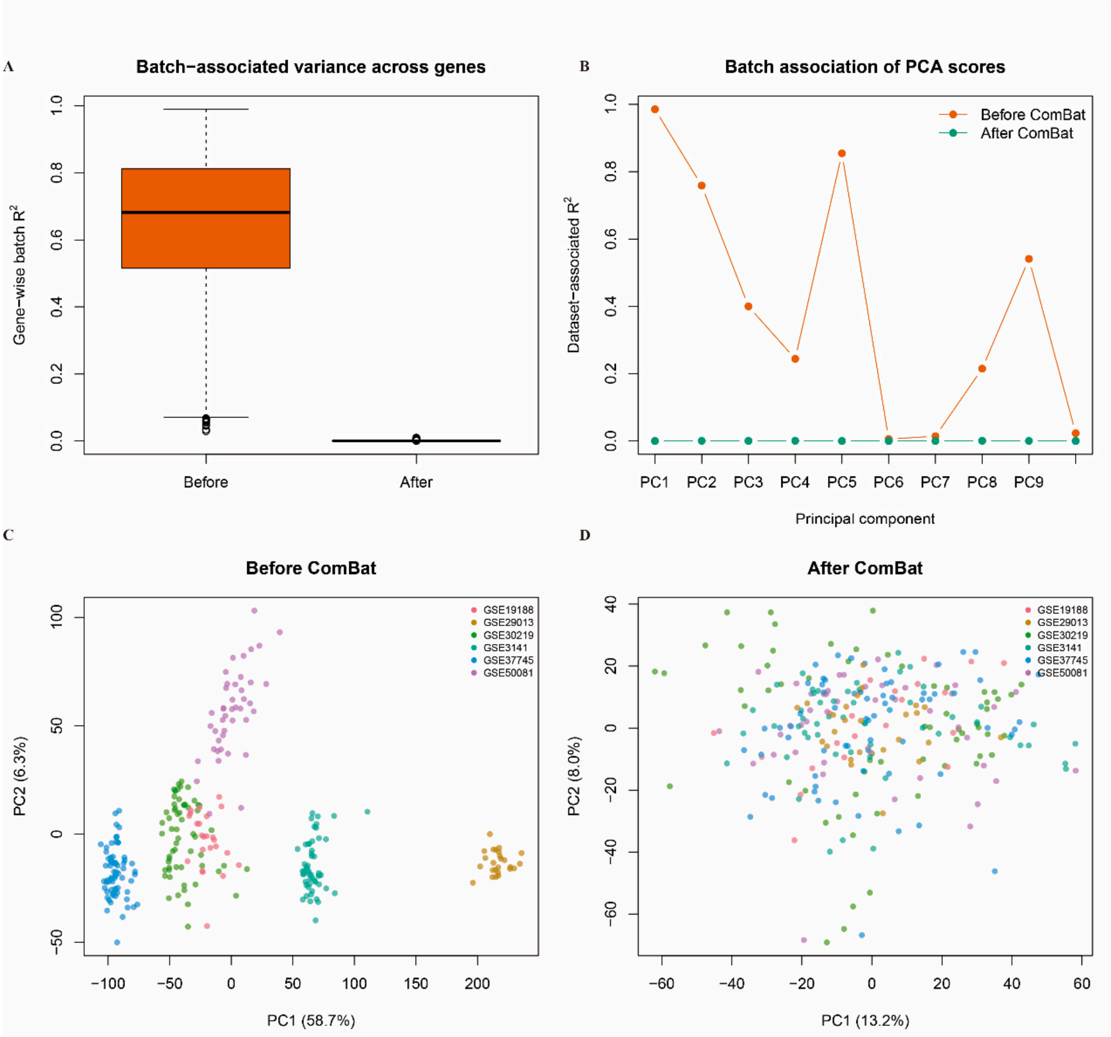

Figure S13. Evaluation of batch effect correction using ComBat in the LUSC cohort.

(A) Distribution of gene-wise batch-associated variance (R^2) before and after ComBat correction. **(B)** Dataset-associated variance (R^2) for the first nine principal components before and after ComBat correction. **(C)** PCA of the merged LUSC cohorts before batch correction. **(D)** PCA of the merged LUSC cohorts after ComBat correction.

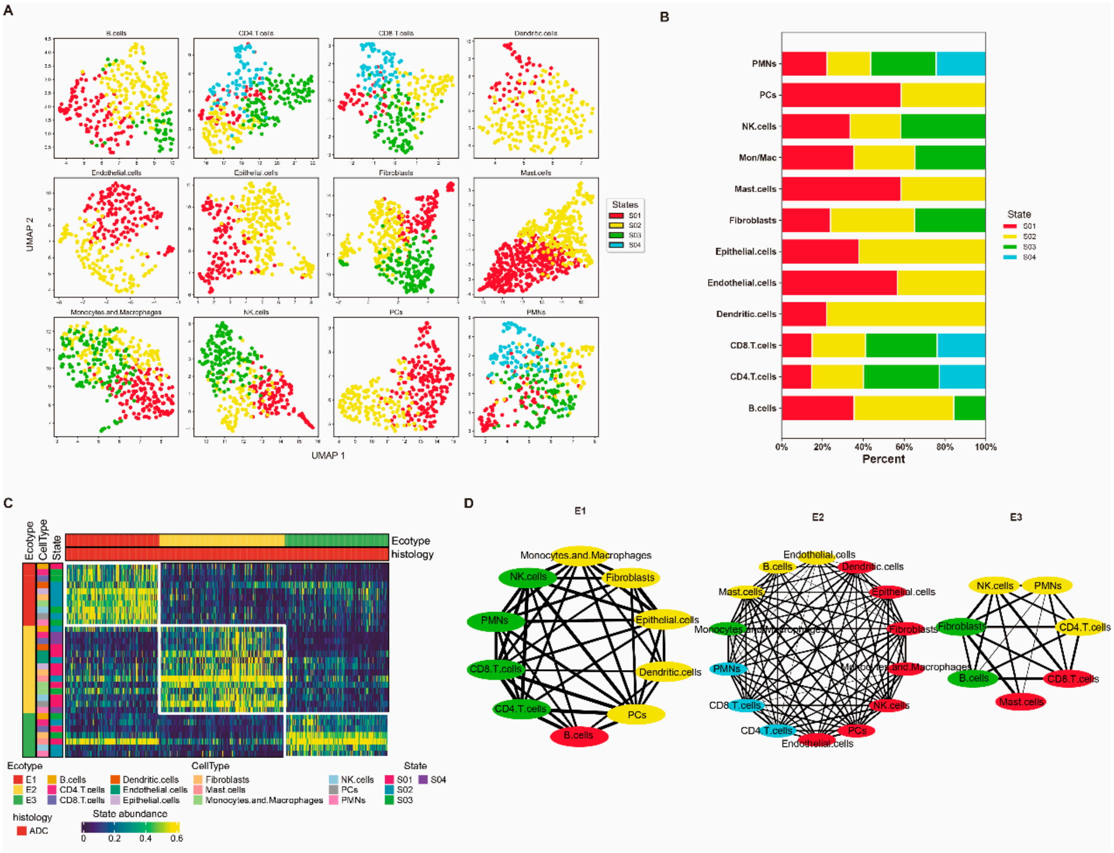

Figure S14. Atlas of transcriptionally defined cell states and multicellular ecotypes in LUAD cohort.

(A) UMAP visualization of 34 cell states identified by the EcoTyper framework across 12 major cell types. Distinct cell states are indicated by different colors.

(B) Bar plots showing the distribution of identified cell states across different cell types. Bars represent the relative proportions of each cell state within individual cell populations.

(C) Heatmap showing the abundance patterns of cell states across six multicellular ecotypes (E1-E3).

(D) Network diagrams showing co-occurrence relationships among cell states within each ecotype. Edge thickness represents the strength of co-occurrence quantified using the Jaccard index.

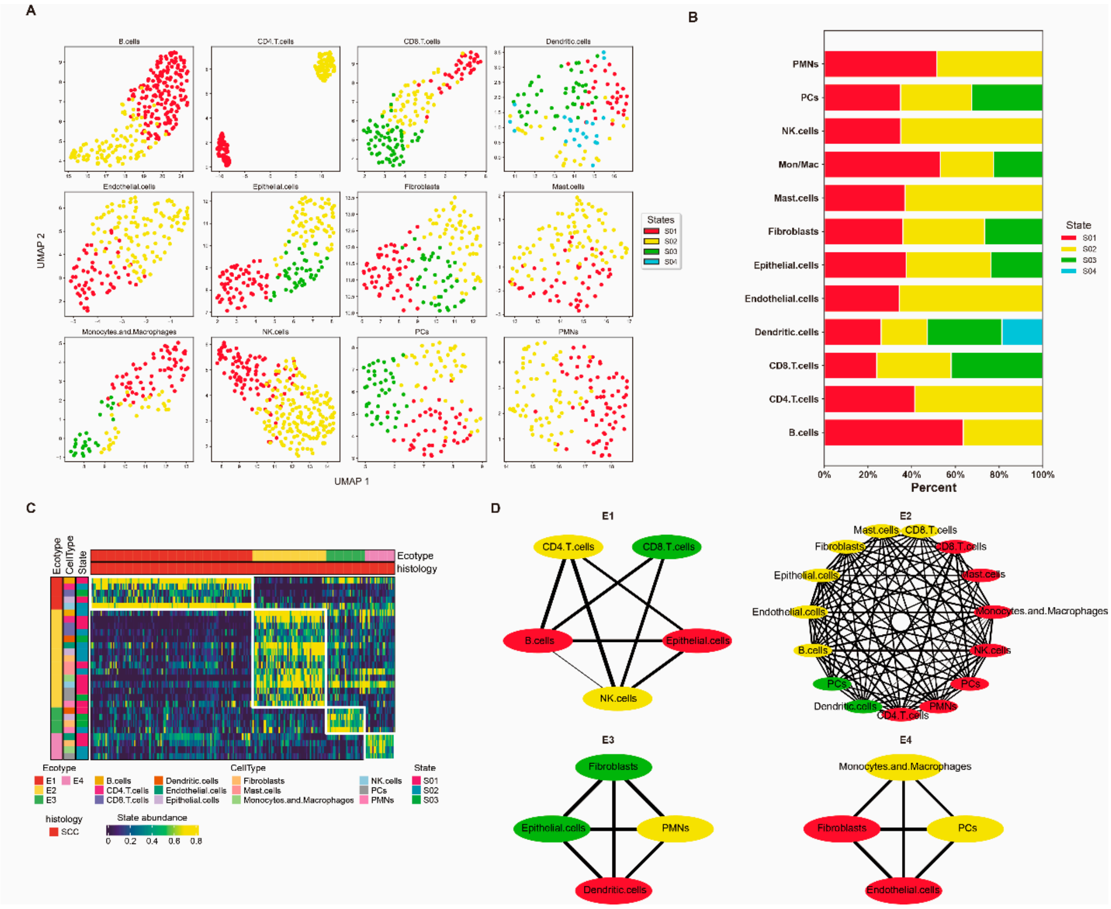

Figure S15. Atlas of transcriptionally defined cell states and multicellular ecotypes in LUSC cohort.

(A) UMAP visualization of 31 cell states identified by the EcoTyper framework across 12 major cell types. Distinct cell states are indicated by different colors.

(B) Bar plots showing the distribution of identified cell states across different cell types. Bars represent the relative proportions of each cell state within individual cell populations.

(C) Heatmap showing the abundance patterns of cell states across six multicellular ecotypes (E1-E4).

(D) Network diagrams showing co-occurrence relationships among cell states within each ecotype. Edge thickness represents the strength of co-occurrence quantified using the Jaccard index.
